# Dissecting Cytophagalysin: Structural and Biochemical Studies of a Bacterial Pappalysin-Family Metallopeptidase

**DOI:** 10.3390/biom14121604

**Published:** 2024-12-16

**Authors:** Eva Estevan-Morió, Juan Sebastián Ramírez-Larrota, Enkela Bushi, Ulrich Eckhard

**Affiliations:** 1 Synthetic Structural Biology Group, Molecular Biology Institute of Barcelona (IBMB), Spanish National Research Council (CSIC), 08028 Barcelona, Spain; 2 Doctorate in Biotechnology, Faculty of Pharmacy and Food Sciences, University of Barcelona, 08028 Barcelona, Spain

**Keywords:** metallopeptidase, pappalysin family, recombinant protein expression, protease activation, functional characterization

## Abstract

Cytophaga is a genus of Gram-negative bacteria occurring in soil and the gut microbiome. It is closely related to pathogenic *Flavobacterium* spp. that cause severe diseases in fish. Cytophaga strain L43-1 secretes cytophagalysin (CPL1), a 137 kDa peptidase with reported collagenolytic and gelatinolytic activity. We performed highly-confident structure prediction calculations for CPL1, which identified 11 segments and domains, including a signal peptide for secretion, a prosegment (PS) for latency, a metallopeptidase (MP)-like catalytic domain (CD), and eight immunoglobulin (Ig)-like domains (D3–D10). In addition, two short linkers were found at the D8–D9 and D9–D10 junctions, and the structure would be crosslinked by four disulfide bonds. The CPL1 CD was found closest to ulilysin from *Methanosarcina acetivorans*, which assigns CPL1 to the lower-pappalysin family within the metzincin clan of MPs. Based on the structure predictions, we aimed to produce constructs spanning the full-length enzyme, as well as PS+CD, PS+CD+D3, and PS+CD+D3+D4. However, we were successful only with the latter three constructs. We could activate recombinant CPL1 by PS removal employing trypsin, and found that both zymogen and mature CPL1 were active in gelatin zymography and against a fluorogenic gelatin variant. This activity was ablated in a mutant, in which the catalytic glutamate described for lower pappalyins and other metzincins was replaced by alanine, and by a broad-spectrum metal chelator. Overall, these results proved that our recombinant CPL1 is a functional active MP, thus supporting the conclusions derived from the structure predictions.

## 1. Introduction

Zinc-dependent metallopeptidases (MP) are ubiquitous peptido- and proteolytic catalysers engaged in a plethora of biochemical pathways [1], and they are subdivided into numerous families and clans. These include the aspzincin [2], gluzincin [3], and metzincin [4] clans, which together with S2P-zincins [5,6], FtsH-like AAA MPs [7], and iron-dependent peptide deformylase [8] form the zincin tribe of MPs [6,9]. Characteristic of zincins is a short motif (H–E–x–x–H; amino-acid one-letter-code, x for any residue), which includes two zinc-binding histidines and a catalytic glutamate. The latter acts first as a general base and then as a general acid during the catalytic mechanism, which pivots around the nucleophilic attack of a zinc-bound catalytic solvent molecule onto the scissile bond of a peptidic substrate bound to the active-site cleft [1,6,9]. Glutamate replacement with alanine hampers catalysis but maintains the active-site geometry [9,10,11,12], so such mutants are often used to create inactive catalytic domains or zymogens for structural and binding studies [9]. The metzincin clan, in turn, is hallmarked by a C-terminal extension of the motif (H–E–x–x–H–x–x–G/N–x–x–H/D), which includes a further glycine or asparagine essential for a conserved turn of the polypeptide chain that leads to a third zinc ligand three positions downstream in sequence, either a histidine or an aspartate [6,9,13,14]. At the structural level, metzincins share a compact ellipsoidal catalytic domain (CD) of ~130–410 residues divided by the active-site cleft into an upper N-terminal subdomain (NSD) and a lower C-terminal subdomain (CSD) when pictured in the consensus standard orientation of MPs [15]. Metzincin NSDs share an “active-site helix”, which encompasses the first half of the extended zinc-binding motif, a “backing helix”, and a four/five-stranded β-sheet, whose lowermost β-strand forms the “upper rim” of the active-site cleft. CSDs share a “C-terminal helix” and a “Met-turn” centred on a conserved methionine residue that creates a hydrophobic base for the active-site zinc.

Within metzincins, the pappalysin family [16] is named after its founding member, human pappalysin-1 or pregnancy-associated plasma protein-A, which is a ~180 kDa multidomain protein first reported from the bloodstream of pregnant women [17,18] whose cryo-electron microscopy structure has been recently elucidated [12,19]. Pappalysins are also grouped within family M43 in the MEROPS peptidase database (www.ebi.ac.uk/merops; accessed on 20 November 2024) [20], and one subfamily features the “lower pappalysins” (LOPAPs) [21], previously referred to as “unicellular pappalysins” [22,23]. LOPAPs encompass similar sequences from archaea, bacteria, and, more recently, also unicellular and multicellular lower eukaryotes like algae and fungi [21]. LOPAPs share at least a short N-terminal prosegment (PS) for zymogenicity, which is a common feature of most MPs [9], and the zinc-dependent metzincin-type CD. Over the years, subfamily members have been described at the transcriptional, functional, and/or biochemical levels (see Table 1 in [21]), among which two have also been analysed for their crystal structures: mirolysin from the periodontopathogenic Gram-negative bacterium *Tannerella forsythia* and ulilysin from the environmental methanogenic archaeon *Methanosarcina acetivorans* [21,22,23,24,25,26,27]. The latter, also known as lysargiNase [21,28], is frequently used in bottom-up proteomics due to its trypsin-mirroring cleavage site specificity.

*Cytophaga* is a bacterial genus that belongs to the *Bacteroidetes* phylum, formerly referred to as the CFB (*Cytophaga*, *Bacteroides*, *Flavobacterium*) phylum [29], which is dominant in gut microbial communities and inhabits the oral cavity, gastrointestinal tract, and urogenital tract of humans [30]. *Cytophaga* sp. strain L43-1, which is closely related to the *Flavobacterium* genus [31], secretes cytophagalysin (CPL1), a 1282-residue multi-modular LOPAP originally purified from culture supernatant and characterized [32]. Moreover, it was cloned and sequenced (see [33,34]) and is listed within the UniProt database (UP) [35] under entry Q46348. The purified enzyme was shown to cleave β-casein, both insoluble and acid-soluble collagens and gelatin, optimally at pH 7.5 and 30 °C, but not small fluorogenic peptides that are cleaved by other bacterial collagenases [34].

Closely related to CPL1, an orthologue (UP A0A2H1E968) is encoded by *Tenacibaculum maritimum* NCIMB 2154 [36], a devastating pathogenic flavobacterium that causes tenacibaculosis in wild and farmed marine fish. Another pathogen, *Flavobacterium psychrophylum*, causes cold-water disease in *Salmonidae* and *Osmeridae* fish and encodes the 960-residue MPs WA-1 and WA-2, which are ~50% identical in sequence to CPL1 [31]. Other potential orthologues are encoded by the environmental bacteria *Sediminitomix flava* (UP A0A315ZEZ8) from marine sediment and *Chitinophaga solisilvae* (UP A0A3S1B1Z0) from forest soil. Importantly, any type of gelatinase or collagenase, including cytophagalysin, represents highly sought-after enzymes for biotechnology. Its ability to degrade collagen makes it valuable in medical tissue engineering through collagen remodelling. Collagenases play a crucial role in environmental bioremediation by degrading collagen- and gelatin-based materials, thereby facilitating their utilization in the leather processing and food production industries.

Here, we aimed to shed light on the structure and function of CPL1 and performed structure-prediction calculations. We analysed these results in the light of the accumulated knowledge on MPs in general and LOPAPs in particular. We further designed CPL1 constructs spanning distinct domains and achieved their recombinant overexpression and purification. Finally, we functionally assessed them in peptide- and proteolytic assays in vitro, and performed mutants to validate their activity.

## 2. Materials and Methods

### 2.1. Biocomputational Studies

Sequence similarity searches and alignments were performed with the *Blast* algorithm [37] within UP and *MultAlin* [38], respectively, using standard parameters. The three-dimensional structure of full-length CPL1 was predicted with *AlphaFold* [39] using paired multiple sequence alignments, which enable the extraction of coevolutionary information and enhance the prediction accuracy [40]. The confidence of the five distinct predictions obtained was assessed by means of the predicted local-distance difference test (pLDDT [41]), which reliably estimates the accuracy of the Cα local-distance difference test [39]. In this respect, pLDDT values >90% account for high accuracy of the overall prediction, and values >70% qualify as generally correct predictions of the backbone [42]. The models were visually inspected for chemical sense and coherence with *Coot* [43]. The top prediction (model_1) was subjected to manual model building to correct clashes and chemical inconsistencies, followed by geometrical regularisation with *Coot*. Thereafter, the *Geometry_minimization* routine of the *Phenix* suite [44] was applied, and the resulting final model, which can be downloaded as part of the Supplementary Materials, was validated with *Molprobity* [45] (Table S1). Inter-domain interfaces were analysed with *Pisa* [46] and structural superpositions were calculated using the *Ssm* routine [47] in *Coot*. Structural relatives present in the Protein Data Bank (PDB) were retrieved through *Dali* [48] and structural figures were prepared with *Chimera* [49] or an open-source build of PyMOL (Version 2.5 Schrödinger, LLC, New York, NY, USA) [50].

### 2.2. Molecular Cloning of CPL1 Constructs

The sequence of full-length CPL1 (Q^20^–K^1282^) from *Cytophaga* sp. L43-1 ^31^ was codon-optimized for expression in *E. coli* (Supplementary Figures S4 and S5), and a synthetic gene was purchased from GenScript Biotech for introduction into a modified pCri7a plasmid ^66^, hereafter pCri7a*, using the *Nde*I and *Bam*HI restriction sites. This plasmid added an N-terminal Strep-tag with a recognition sequence for tobacco-etch virus peptidase (TEV) and a C-terminal His_8_-tag. Three C-terminally truncated variants of CPL1, designated CPL1_1-2 (Q^20^–N^328^), CPL1_1-3 (Q^20^–V^444^), and CPL1_1-4 (Q^20^–T^591^), were subsequently cloned by inverse PCR [51] using 0.5 µM of forward and reverse primers, 200 µM of each of the four dNTPs, 0.02 U/µL Q5 High-Fidelity DNA Polymerase (New England Biolabs), but no High GC Enhancer. Constructs were produced as wild-type species and as mutants, in which the catalytic glutamate from the zinc-binding motif had been replaced with alanine (E^232^A). The two variants of CPL1_1-3 and CPL1_1-4 were also introduced into plasmid pCri7b [52], which only adds the C-terminal His_8_-tag, using the same restriction sites. Cloning and mutagenesis primers are listed under Supplementary Figure S6A,B, and the map of pCri7a* is shown under Supplementary Figure S6C.

### 2.3. Protein Expression and Purification

Various chemically competent *E. coli* strains, including BL21(DE3), Origami 2(DE3), Lemo21(DE3), and Rosetta(DE3), were prepared inhouse following the Inoue method [53], transformed with the different protein-encoding plasmids, and plated on agar plates containing Bertani’s lysogeny broth [54] supplemented with kanamycin at 50 μg/mL (hereafter medium). After overnight incubation, a single colony was picked for each strain and plasmid, and cultured in 5 mL medium at 37 °C under gentle shaking (at 200 rpm). Cultures were then diluted 1:1000 in 500 mL fresh medium using four 2 L Erlenmeyer flasks, grown at 37 °C to OD_600_ ≈ 1.2, cooled for 15–30 min in a cold room, induced with 1 mM isopropyl-β-D-1-thiogalactopyranoside (IPTG), and placed at 20 °C under gentle shaking for overnight protein expression. Cells were subsequently harvested by centrifugation, resuspended in 30 mL buffer A (50 mM Tris·HCl pH 8.0, 300 mM sodium chloride, 10 mM imidazole, 10 mM calcium chloride, 50 µM zinc chloride), and sonicated on ice for 1 min at 20% amplitude and 0.5 s intervals using a Branson 450 Digital Sonifier (Marshall Scientific, Hampton, NH, USA) with a 6 mm tapered microtip. The resulting solution was incubated on ice with magnesium chloride at 10 mM and 10 µg/mL DNase I for 15 min, and then lysed using an E1061 continuous flow cell disruptor (Constant Systems, Daventry, UK) operated at 1.36 kbar. Lysates were incubated for 30 min on ice with 1 M urea before being centrifuged twice at 20,000 rpm using a JA-25.50 rotor in a Beckman Avanti J-25 centrifuge (~48,000× *g*). The clarified supernatants were subjected to affinity chromatography employing HisPur Ni-NTA resin (Thermo Scientific, Waltham, MA, USA) and buffer A plus 20 mM or 250 mM imidazole for the wash or elution steps, respectively. Eluted samples were then concentrated using a Vivaspin-20 polyethersulfone centrifugal device with 10 kDa molecular-weight cutoff (Sartorius, Göttingen, Germany), and polished by SEC in Superdex 200 10/300 GL or Superose 6 Increase 10/300 GL columns (Cytiva, Marlborough, MA, USA), previously equilibrated with buffer B (50 mM Tris·HCl pH 8.0, 100 mM sodium chloride) and attached to an ÄKTApurifier 10 protein purification system (GE Healthcare, Milwaukee, WI, USA). Peak fractions were pooled, re-concentrated by centrifugation, and flash-frozen in liquid nitrogen as 50 μL and 10 μL aliquots of 10 mg/mL and 1 mg/mL concentration, respectively, and stored at −80 °C until usage. Protein concentrations were determined using a BioDrop Duo+ microvolume spectrophotometer (BioChrom, Cambridge, UK) based on the absorbance measured at λ = 280 nm and the respective theoretical extinction coefficient calculated by *ProtParam* [55,56]. Protein purity was assessed by SDS-PAGE analysis on custom-made 12% and 14% Tris/Glycine gels followed by Coomassie Brilliant Blue (Sigma-Aldrich, Saint Louis, MO, USA) staining. The BlueStar Plus Prestained protein marker (Nippon Genetics EUROPE, Düren, Germany) was used as molecular-mass reference.

### 2.4. Activation of CPL1

For activation, the wild-type CPL1 constructs, which encompass the predicted PS for latency (Q^20^–A^66^; see Section 3.2), were incubated with bovine trypsin, treated with *N*-tosyl-L-phenylalanyl chloromethyl ketone (TPCK) to prevent extraneous chymotryptic activity (Sigma-Aldrich, Saint Louis, MO, USA), at a weight ratio of 1000:1 for 10–15 min at room temperature, and activation through PS removal was monitored by SDS-PAGE. Similarly, bovine α-chymotrypsin, treated with *N*-tosyl-L-lysinyl chloromethyl ketone (TLCK) to ablate undesired trypsin activity (Sigma-Aldrich, Saint Louis, MO, USA), was likewise used to assess activation. In either case, reactions were stopped by adding the EDTA-free HALT inhibitor cocktail (Thermo Fisher Scientific, Waltham, MA, USA) at a final concentration of 2×, which is equivalent to 2 mM of the broad-spectrum serine peptidase inhibitor 4-(2-aminoethyl)benzenesulfonyl fluoride (AEBSF).

### 2.5. Activity Assessment by Zymography

Activated CPL1 variants were loaded onto 12% or 14% SDS-PAGE gels containing 0.1% gelatin from cold water fish skin (Sigma-Aldrich) and subjected to electrophoresis at 160 V for ~1 h on ice. Thereafter, gels were extensively rinsed with distilled water and washed three times for 30 min in buffer C (50 mM Tris·HCl pH 8.0, 150 mM sodium chloride, 2.5% Triton X-100) under gentle shaking. After two further washes with buffer D (50 mM Tris·HCl pH 8.0, 150 mM sodium chloride, 10 mM calcium chloride, 50 μM zinc chloride, 0.02% Brij-35) for 20 min, gels were incubated in the same buffer overnight at room temperature, and stained with Coomassie Brilliant Blue. After a brief destaining step, proteolytic activity was revealed as light bands against over a dark blue background. All samples for zymography were prepared with non-reducing sample buffer without heating if not stated otherwise.

### 2.6. Fluorometric Gelatinolytic Activity and Inhibition

Activity of CPL1 variants against gelatin in solution was determined using the substrate DQ Gelatin from pig skin (Thermo Fisher Scientific, Waltham, MA, USA), which consists of highly quenched fluorescein-labelled gelatin that yields bright green fluorescence upon proteolysis of the substrate. Substrate at 20 μg/mL in 100 μL buffer E (50 mM Tris·HCl pH 8, 150 mM sodium chloride, 10 mM calcium chloride, 50 µM zinc chloride, 1× Halt^TM^ Protease Inhibitor Cocktail; Thermo Fisher Scientific, Waltham, MA, USA) was reacted with CPL1 at 37 °C, and the fluorescence response was monitored in a BioTek Synergy H1 multimode microplate reader (Agilent Technologies, Santa Clara, CA, USA) with λ_exc_ = 480 nm and λ_em_ = 520 nm. Activity inhibition was assessed by preincubating the CPL1 variants with 20 mM EDTA for 30 min and proceeding as mentioned.

### 2.7. Collagen Degradation Assay

Activity of CPL1 variants against the VitroCol type-I human atelocollagen (Advanced Biomatrix, Carlsbad, CA, USA) and against rat-tail type-I collagen (Corning, Corning, NY, USA) was tested in 40 µL reaction volumes by adding 2 µg of enzyme to 10 µg of collagen in buffer E. After 16 h of incubation at 20 °C to ensure native collagen conditions, cleavage was assessed by 12% SDS-PAGE gels. Collagenase type IA from *Clostridium histolyticum*, bovine TPCK-treated trypsin, and bovine TLCK-treated α-chymotrypsin (all from Sigma-Aldrich, St. Louis, MO, USA) were used as controls.

### 2.8. Protein Crystallization Assays

Crystallization assays were performed by the sitting-drop vapor diffusion method. Reservoir solutions were prepared by a Tecan robot and 100 nL crystallization drops were dispensed on 96-well 2-drop Swissci PS MRC plates (Molecular Dimensions, Sheffield, UK) by a Phoenix nanodrop robot (Art Robbins Instruments, Sunnyvale, CA, USA) or a Cartesian Microsys 4000 XL robot (Genomic Solutions, Ann Arbor, MI, USA) at the joint IBMB/IRB Automated Crystallography Platform (https://ibmb.csic.es/en/platforms/automated-crystallographic-platform; accessed on 20 November 2024). Plates were stored in steady-temperature crystal farms (Bruker, Billerica, MA, USA) at 4 °C or 20 °C and regularly inspected.

## 3. Results and Discussion

### 3.1. Computational Prediction of the CPL1 Structure

**Figure 1:**
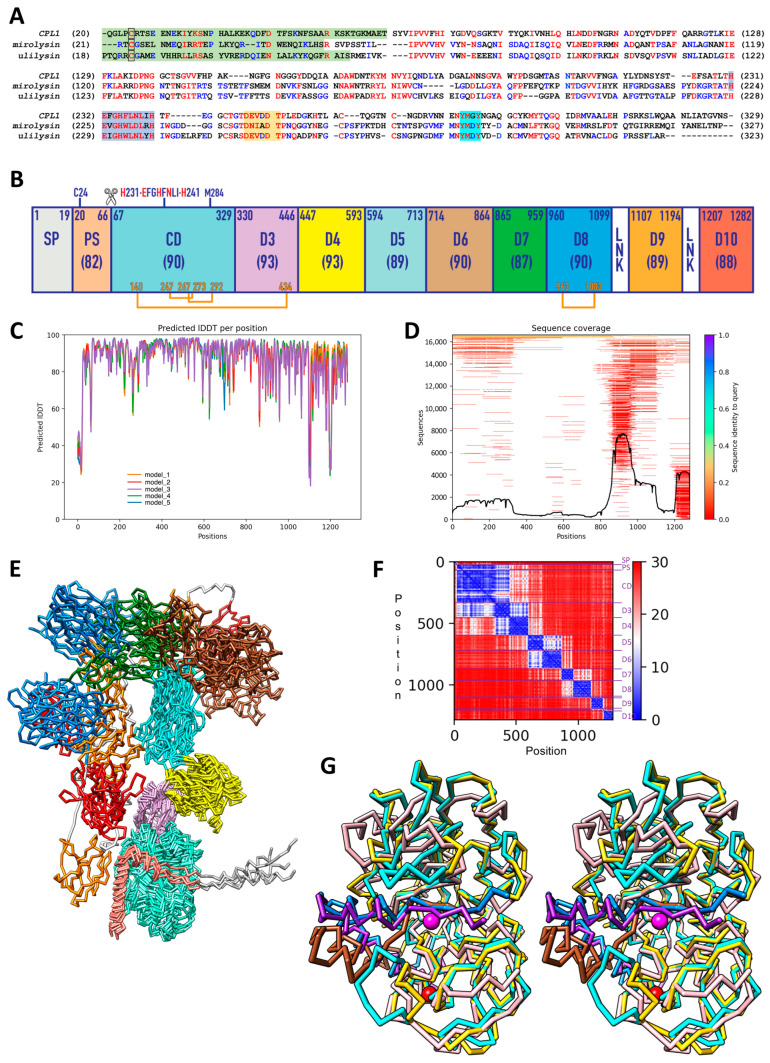
Biocomputational studies. (**A**) Sequence alignment of the prosegments (PSs) (green background) and catalytic domains (CDs) of CPL1 (UP Q46348), mirolysin (UP G8ULV1), and ulilysin (UP Q8TL28). Identical or equivalent residues are in red, and those shared by two sequences are in blue. The PS cysteine engaged in zinc-binding in the zymogen—putatively in CPL1—is framed. The extended zinc-binding motif, the residues shaping the common calcium site, and the Met-turn motif are shown over light-blue, orange, and cyan background, respectively. (**B**) Domain distribution along the chemical sequence predicted by *AlphaFold*, which foresees a signal peptide for secretion (SP), the PS, the CD, and immunoglobulin-like domains D3 through D10. Each domain is labelled, the respective limiting residues are indicated, and the average predicted local-distance difference test (pLDDT) is shown in parenthesis. In all cases, these values are close to or exceed the high-accuracy cut-off of ~90% [42], and are thus classed as high confidence. The only exception is the PS, whose prediction evinces an average pLDDT that is slightly lower, but still highly reliable for the main chain. Two short linkers (LNKs) would be intercalated between D8 and D9, and between D9 and D10. Predicted disulfide bonds are shown in orange. The cysteine putatively engaged in latency in the zymogen (C^24^) and the extended zinc-binding motif (H^231^–H^241^), as well as the Met-turn methionine (M^284^) and the maturation cleavage point (A^66^–E^67^; scissors) are further pinpointed. (**C**) pLDDT for each residue of the prediction (positions 1–1282) for each of the five distinct models obtained. (**D**) Sequence coverage for each residue of the prediction (positions 1–1282) vs. number of sequences. (**E**) Superposition of the five predicted models without further relaxation/minimization with each domain/segment in the colour of (**B**). Only PS, CD, D3, D4, and, roughly, D5 appear with similar relative orientations in all models. (**F**) Analysis of the predicted aligned error, which estimates if domains are correctly positioned relative to one another, for each residue of the prediction (positions 1–1282; model_1). Each segment/domain of (**B**) gives rise to a marine blue square along the diagonal. Off-diagonal blue values suggest well-predicted interactions between domains. (**G**) Superposition of the Cα-traces of the experimental structures of promirolysin (PS in sienna, CD in gold) and proulilysin (cyan/dodger blue) in standard orientation [15] onto the prediction of CPL1 (purple/pink). The CPL1 prediction matches proulilysin significantly better. The catalytic zinc (magenta sphere) and the common calcium (red sphere) of proulilysin are further displayed.

Similarity searches identified mirolysin (UP A0A0F7IPS1 and G8ULV1, wrongly entitled “karilysin”) and ulilysin (UP Q8TL28) as the closest sequences of CPL1 among structurally analysed proteins (*p* = 1.8 × 10^−23^, 269 common residues, 30% sequence identity; and *p* = 1.5 × 10^−20^, 237 common residues, 31% sequence identity, respectively). The aligned sequence stretches encompassed in both cases the respective PSs and CDs, which supports the ascription of CPL1 CD to the LOPAPs (Figure 1A).

Next, we performed computational structure predictions for residues 1–1282 with the *AlphaFold* program [39], which were highly confident according to the predicted local-distance difference test (pLDDT) and sequence coverage (Figure 1C,D). The highest-ranking model of five predictions was subjected to manual rebuilding and geometric minimization. Supplementary Table S1 depicts the statistics of the validation of the resulting working model, which can be downloaded as part of the Supplementary Information. This model proposes the presence of 11 segments and domains plus two linkers, which include a 19-residue signal peptide for secretion (SP) followed by a 47-residue PS (Q^20^–A^66^; sequence numbering of CPL1 in superscript, see UP Q46348) (Figure 1B) that is absent from the enzyme purified from culture supernatant [32,33,34]. Next, the metal-dependent CD (E^67^–S^329^) would contain two intradomain disulfides (C^247^–C^273^ and C^267^–C^292^) plus a third one (C^140^–C^434^) that links this domain to downstream domain D3 (T^330^–P^446^). The latter precedes seven further domains (D4, Y^447^–M^593^; D5, T^594^–S^713^; D6, P^714^–G^864^; D7, I^865^–A^959^; D8, S^960^–G^1099^; D9, T^1107^–P^1194^; and D10, K^1207^–K^1282^), with two short linkers inserted at the D8–D9 and D9–D10 junctions (Figure 1B).

With respect to the relative orientations of the domains, only the first five domains consistently appeared in a comparable arrangement in the five predictions (see Figure 1E). This is consistent with the fact that relative orientations of domains cannot usually be predicted accurately [57]. Indeed, analysis of the predicted aligned error, which accounts for the residue–residue alignment confidence and, thus, if domains are reliably positioned [58], suggests that PS–CD, CD–D3, as well as D3–D4 and D4–D5 might be correctly placed relative to one another in the predictions (Figure 1F). Visual inspection and computational quantification of the inter-domain interfaces with *Pisa* [46] suggested that the relative arrangement of PS–CD and CD–D3 might actually be significant. It features interfaces spanning 1329 Å^2^ and 518 Å^2^, respectively, which are in the range of values reported for experimental protein–protein complexes (955 ± 380 Å^2^) [59], and the calculated Δ^i^G values are −12.2 kcal/mol and −8.9 kcal/mol, respectively. In contrast, the D3–D4 and D4–D5 interfaces feature only few contacts and may rather represent hinge points for structure rearrangement. Future studies may include multi-state small-angle X-ray scattering (SAXS) profile analysis of near-to-full-length protein preparations to experimentally capture the structural interplay of accessory domains.

### 3.2. Comparison with Proulilysin and Promirolysin

**Figure 2:**
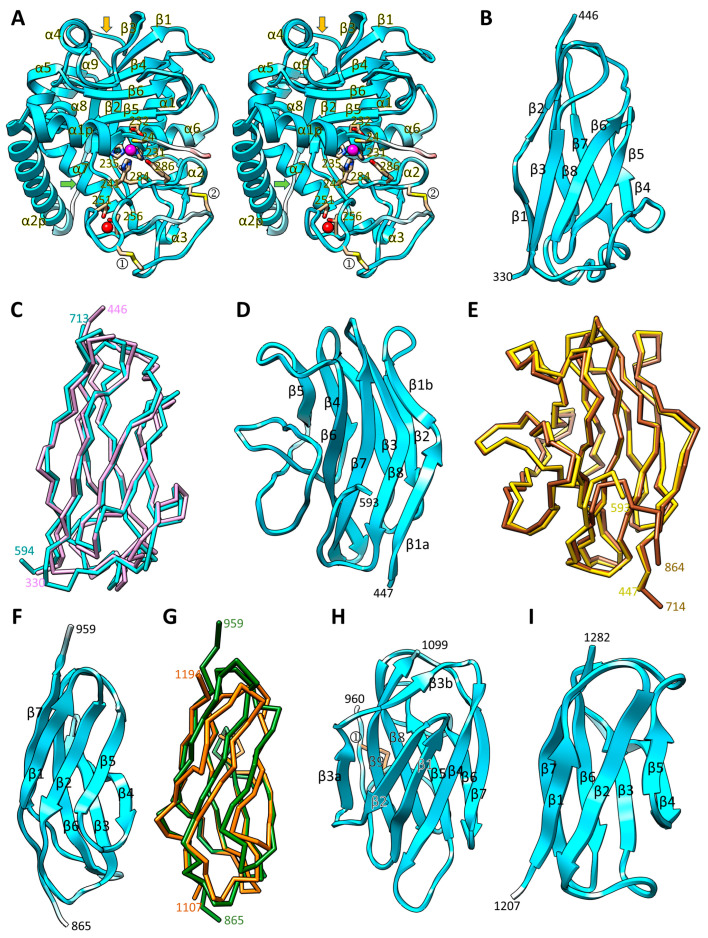
Structural analysis of the predicted CPL1 domains. (**A**) Ribbon-type plot of the CPL1 PS and CD in cross-eye stereo. The secondary structure elements are labelled (α1p, α2p, α1–α9, and β1–β6). The putative cysteine-switch cysteine (C^24^), zinc-binding residues (H^231^, H^235^ and H^241^), general base/acid glutamate (E^232^), Met-turn methionine (M^284^) and tyrosine-switch tyrosine (Y^286^), calcium-binding residues (D^251^ and T^256^), as well as the putative disulfide-bonded cysteines (C^247^–C^273^; ① and C^267^–C^292^; ②) are shown for their side chains as sticks and numbered. The zinc and calcium cations were modelled based on the proulilysin (PDB 8CDB) and mature ulilysin (PDB 2CKI) structures. The putative maturation site (A^66^–E^67^) and the LNR-loop are highlighted by green and orange arrows, respectively. Depiction of the Ig-like domains (D3–D10) showing as ribbon- or Cα-plots (**B**) D3; (**C**) D5 (cyan Cα-plot) onto D3 (plum Cα-plot) in the same orientation as in (**B**); (**D**) D4; (**E**) D6 (brown Cα-plot) onto D4 (yellow Cα-plot) in the same orientation as (**D**); (**F**) D7; (**G**) D9 (orange Cα-plot) onto D7 (green Cα-plot) in the same orientation as (**F**); (**H**) D8 (disulfide bond C^963^–C^1083^; ①) and (**I**) D10. The β-strands and the N- and C-terminal residues are numbered in all cases.

Superposition of the experimental LOPAP structure of *Tannerella* promirolysin (Protein Data Bank [PDB] access code 6R7V [23]) onto the CPL1 working model revealed 213 aligned residues (out of 307 of promirolysin), with a core *rmsd* of 2.7 Å and a sequence identity of 30%. The same calculations employing *Methanosarcina* proulilysin (PDB 8CD8 [21]) revealed 248 aligned residues (out of 306 proulilysin residues) deviating 1.7 Å (29% identity). Overall, the three structures accurately match and share the common structural elements of LOPAPS (Figure 1A,G and Figure 2A). However, the differences in the *rmsd* values point to CPL1 being closer to ulilysin than to mirolysin.

In all structures, the PS runs across the cleft of the CD in the opposite direction of a substrate, which prevents autolytic cleavage, and encompasses two large, characteristic helices (α1p and α2p). This segment blocks access of substrates to the active-site cleft, thus keeping the zymogens inactive. The helices deviate in the *Tannerella* zymogen when compared with the other two structures, which accurately match (Figure 1F). Moreover, C^24^ of CPL1’s PS would be topologically equivalent to C_23_ of both mirolysin and ulilysin (other proteins’ positions are numbered in subscript), which have been shown to play a role in latency in these enzymes by acting as a “cysteine switch” [21,23]. The cysteine Sγ atom blocks the catalytic zinc ion as also described for other metzincins, like matrix metallopeptidases, bacterial astacins, adamalysins, and a-disintegrin-and-metallopeptidase enzymes (ADAMs) [60,61,62,63]. Furthermore, the experimental activation cleavage site of CPL1 (A^66^–E^67^) [32,33], which removes the PS and yields the mature, competent CD (Figure 1A,G), would be topologically equivalent to those of the archaeal (S_60_–R_61_) and bacterial (S_54_–R_55_) relatives [21,23].

As to the CD (Figure 1G and Figure 2A), CPL1 would share with the two LOPAPs the five-stranded β-sheet (top to bottom, β2+β3–β4–β6–β5 in CPL1) including the “LNR-like loop” (L^131^–G^143^), which protrudes from the molecular surface and divides the second strand of the sheet in two (β2+β3). Next, the active-site helix (α6) and the backing helix (α1), as well as the two short helices (α2 and α3) after the latter, which form a cape in the back of the molecule, would be shared. Further common elements are the Met-turn, the C-terminal helix (α8), and two additional α-helices (α8 and α9) after the C-terminal helix, which are characteristic of LOPAPs but not of metzincins in general. Moreover, the active site centred on the catalytic zinc ion and the arrangement of the residues encompassing the extended zinc-binding motif would be very similar, which further supports that CPL1 is actually an MP (Figure 2A) (see also Section 3.5). In addition, the predicted CD disulfides C^247^–C^273^ and C^267^–C^292^ would be topologically equivalent to C_250_–C_277_ and C_269_–C_297_ of ulilysin and C_243_–C_271_ and C_262_–C_291_ of mirolysin. Furthermore, Y_286_ (promirolysin) and Y_292_ (proulilysin), which are found two positions downstream of the Met-turn methionine (Figure 1A), have been reported to play a role in substrate binding during catalysis, acting as a “tyrosine switch” [10]. This residue would also be present in CPL1 as Y^286^ (Figure 2A), likely exerting similar functions. Finally, similarly to proulilysin, the CPL1 zymogen would lack one of two calcium-binding sites, which exert essential structural functions in the mature structures [21,22,23,24,25]. In contrast, this calcium site is already present in the promirolysin zymogen [23]. As to the second site, the model adopts a very similar trajectory to the experimental structures for the protein chain involved (D^251^–T^256^; Figure 1G), thus supporting this site would also be present in CPL1.

Unique for CPL1, an “adamalysin helix” (α4) would be inserted between β3 and β4, nestling on the convex face of the β-sheet (Figure 2A). Such a helix was first reported for adamalysins/ADAMs [64] and then for fragilysin-3 [65,66], and it is replaced in ulilysin and mirolysin by an irregular loop [22,23]. Moreover, an additional helix (α5), which is missing in the other LOPAPs, would be inserted between sheet strands β5 and β6.

### 3.3. Eight C-Terminal Immunoglobulin-like Domains (D3–D10)

Downstream of the CD, domain D3 would adopt a compact immunoglobulin-like (Ig-like) fold [67,68,69], which consists of 117 residues forming an antiparallel β-sandwich with four-stranded front and back sheets (β1–β3–β6–β5 and β2–β8–β7–β4, respectively, from left to right in Figure 2B), with an intersheet angle of ~35° and Greek-key topology. The predicted model of the domain after next (D5) encompasses 120 residues and superposes very accurately onto D3, with 114 aligned residues overlapping with a core *rmsd* of 1.2 Å (Figure 2C). The two domains have 37% sequence identity, so they might be functionally equivalent. Notably, Ig-like domains also represent an intriguing scaffold for the de novo design of antibody-like structures with superior biophysical properties [70,71].

With respect to function, D3 and D5 best match domain Ig-2 of the high-molecular-mass chitinase ChiW from *Paenobacillus* sp. (*rmsd* 3.0 Å; Z-score 9.1 according to [48]; PDB 5GZT [72]). Like D3, this domain is also immediately downstream of and attached to a CD, in this case a carbohydrate hydrolase moiety. Both models share the overall architecture, topology, and connectivity, but not the detailed chain trace, as revealed by the rather high *rmsd* value. Domain Ig-2 and others alike have been proposed to be linkers that connect CDs—or these with substrate-binding domains—and stabilize them within large multi-modular enzymes [72]. The function of D3, and by extension D5, in CPL1 could be similar. Indeed, in the probably most reliably predicted relative arrangement between domains of the working model (see Section 3.1), D3 would interact through its lateral sandwich surface framed by strands β2 and β8 with the LNR-like loop of the preceding CD. This interaction would be cemented through a disulfide bond (C^140^–C^434^) that is unique for LOPAPs.

Linked to D3, the 147-residue D4 domain would likewise be an antiparallel 4+4 β-sandwich (Figure 2D) with a front sheet, in which the first strand is halved by a bulge (from left to right, β6–β7–β8–β1a+β1b), and a back sheet (β5–β4–β3–β2). This domain differs from D3/D5 in the topology of the β-sheets. In addition, the difference in size is accounted for by an 11-residue C-terminal extension and a wide 41-residue spiral segment, which connects strands β4 and β5 and laterally contacts the left sandwich surface framed by strands β5 and β6 (Figure 2D). This confers on D4 a much more globular and bulky shape than D3. Next-but-one domain D6, which encompasses 151 residues and is thus the largest of the D3–D10 series, superposes very accurately onto D4, with 147 aligned residues overlapping with a core *rmsd* of 0.8 Å (Figure 2E). The two domains have a remarkable 63% sequence identity, so, as the preceding D3/D5 tandem, they are most likely functionally equivalent.

Domains D4/D6 do not really have close structural relatives. Merely the overall topology and connectivity of the β-sandwich is shared with a haeme-binding protein from *Tannerella forythia* (*rmsd* 3.5 Å; Z-score 7.1; 8% sequence identity; PDB 6EU8 [73]) and a putative lipoprotein from *Bacteroides fragilis* of unknown function (*rmsd* 3.1 Å; Z-score 6.0; 9% sequence identity; PDB 4GBS [74]). However, these proteins are substantially larger (192 and 186 residues) and possess several additional structural elements. Thus, no hint on a possible function for D4/D6 can be anticipated at this point.

Domain D7 (Figure 2F) would be an elongated canonical 95-residue Ig-like fibronectin type-III domain (FNIII), as inferred from an accurate match (*rmsd* 1.7 Å) with the eponymous 92-residue domain from the thermophilic bacterium *Thermoanaerobacter tengcongensis* (Z-score 13.9; PDB 7JGT [75]). The prediction evinces an antiparallel three-stranded (β1–β2–β5) β-sheet plus a four-stranded (β7–β6–β3–β4) β-sheet arranged in a β-sandwich. The sheets have an intersheet angle of ~45° and they follow a Greek-key topology. Overall, this fold is reminiscent of the 90-residue third FNIII domain of tenascin (PDB 1TEN [76]) and is prototypic for the s-type Ig-like fold according to Ref. [67]. Similarly to the D3/D5 and D4/D6 tandems, the next-but-one domain D9 of 88-residues adopts the same fold as D7 in the prediction (Figure 2G), as revealed by 85 aligned residues overlapping with a core *rmsd* of 1.8 Å. The two domains have 24% sequence identity, so they might have an equivalent function. FNIII domains are found in cell-adhesion molecules, muscle proteins, in enzymes, and in extracellular matrix proteins, but they have no common function [77].

Domain D8 would likewise adopt a compact Ig-like fold, with 140 residues forming an antiparallel β-sandwich with a four-stranded front sheet (β2–β1–β4–β7 in Figure 2H) and a five-stranded back sheet, in which the leftmost strand is halved by a bulge (β3a+β3b–β9–β8–β5–β6). The sheets have an intersheet angle of ~35° and they follow a Greek-key topology. The strands are connected by short loops except for strands β8 and β9 of the back sheet, which are connected by an 11-residue flap that folds back and lines the back surface of the sheet. This surface is further formed by a segment in extended conformation preceding the first strand of the front sheet, which is connected to the flap through a disulfide bond (C^963^–C^1083^; Figure 2H). The D8 domain is reminiscent of a guanine nucleotide dissociation inhibitor of rho G proteins [78,79], which was the closest match in structure similarity searches after visual inspection (PDB 1DOA; Z-score 7.9). Overall, this architecture encompasses the 3+4-stranded s-type Ig-like fold according to Ref. [67] found in D7/D9, but it contains extra β-strands and loops, as also found in the receptor-binding domain of mammalian α_2_-macroglobulin [80,81,82].

Finally, the prediction of domain D10 (Figure 2I) presents the same three-stranded front β-sheet and four-stranded back β-sheet architecture and connectivity as D7/D9 (β1–β2–β5 and β7–β6–β3–β4), but it is significantly smaller (76 residues) and more compact, so that superposition onto the former domains yields a poor match. Instead, it fits the 65-residue C-terminal domain (CTD) of gingipain RgpB (PDB 5AG8 [83]; Z-score 8.4) much better. This moiety is the outer-membrane export signal of the type-IX secretion system (T9SS) of the periodontopathogenic Gram-negative bacterium *Porphyromonas gingivalis* [84,85,86], which exports several virulence factors to the outer membrane and the extracellular space. Similar T9SSs have been found in several other *Bacteroidetes* [85] including *Cytophaga hutchinsonii* [87,88], which is predicted to secrete at least 147 proteins [89]. These include enzymes and other molecules that degrade cellulose and other polysaccharides on the cell surface [89,90]. Moreover, the CPL1 orthologues WA-1 and WA-2 from *F. psychrophylum* have also been reported to possess a CTD for T9SS export [31]. Thus, the structural similarity of D10 with CTDs suggests a similar role for the former, which is also the C-terminal domain of a multi-domain protein, in the export across the outer membrane. It remains to be experimentally determined if CPL1 is a cargo protein of the *Cytophaga* T9SS.

### 3.4. Recombinant Production of CPL1

In order to gain experimental insight into the molecular determinants suggested by the aforementioned modelling studies, we designed various CPL1 constructs for recombinant overexpression and functional analysis. These included, in addition to the full-length form including domains PS+CD+D3–D10 (Q^20^–K^1282^), further CPL1_1-2 spanning PS+CD (Q^20^–N^328^), CPL1_1-3 encompassing PS+CD+D3 (Q^20^–V^444^), and CPL1_1-4 featuring PS+CD+D3+D4 (Q^20^–T^591^). We considered two variants for each construct, the wild-type and a mutant, in which the catalytic glutamate had been replaced with alanine (E^232^A-mutant), thus yielding the protein constitutively inactive (see Section 1). To this aim, we employed variants of two recombinant expression vectors (pCri7a and pCri7b) [52], which provide removable and non-removable tags for nickel-affinity chromatographic purification, as well as a total of four *E. coli* strains with different properties (Section 2.3). In all cases, proteins were subjected to a final polishing step by size-exclusion chromatography (SEC).

**Figure 3:**
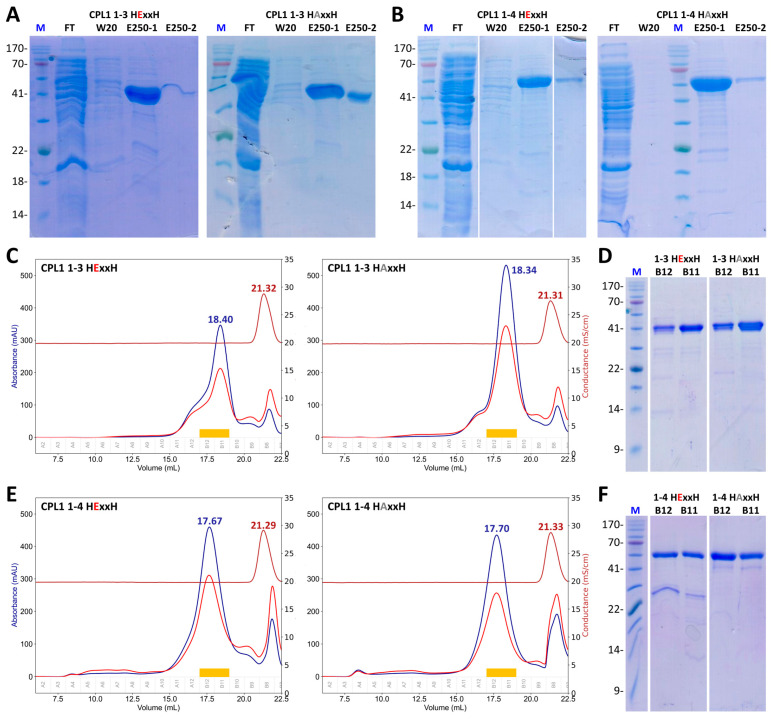
Recombinant CPL1 expression and purification. (**A**) Representative SDS-PAGE gels of nickel-affinity purifications of construct CPL1_1-3 (Q^20^–V^444^), both in its wild-type (left panel) and E^232^A-mutant (right panel) variants. Samples representing the flow through (FT), the wash-step with 20 mM imidazole (W20), and the first two elution fractions using 250 mM imidazole (E250-1/2) were analysed under reducing conditions. Lane M depicts the molecular-mass marker. The target protein migrated as a band at its expected molecular weight (~47 kDa). (**B**) Same as (**A**) for construct CPL1_1-4 (Q^20^–T^591^), which migrates as a ~63 kDa band as expected. (**C**) Representative calibrated size-exclusion chromatography profiles of the two constructs of (**A**) using bovine-serum albumin as the calibration standard, and with the conductivity trace shown in dark red (peak at ~21.3 mL), and (**D**) SDS-PAGE analyses of peak fractions B11 and B12 shown in (**C**) as orange bands. A retention volume of ~18.4 mL corresponds to an apparent molecular mass of ~41 kDa, which is consistent with the theoretic value (~47 kDa). (**E**,**F**) Same as (**C**,**D**) for the two constructs of (**B**). A retention volume of ~17.7 mL corresponds to an apparent molecular weight of ~61 kDa, which is consistent with the theoretic value (~63 kDa). SDS-PAGE gels were cropped for clarity. For full gel images, please refer to Extended Data Figures S7–S10 in the supplement.

Despite extensive trials, we did not succeed in obtaining well-folded protein in amounts large enough for biochemical studies for the full-length form (Supplementary Figure S1). In contrast, we obtained substantial amounts of highly pure preparations of both types of variants for CPL1_1-3 (Figure 3A,C,D and Supplementary Figure S2A) and CPL1_1-4 (Figure 3B,E,F and Supplementary Figure S2B), as well as minute amounts of wild-type CPL1_1-2 for some tests (Supplementary Figure S2C). CPL1_1-3 and CPL1_1-4 migrated in calibrated SEC according to their theoretical molecular masses (Figure 3C,E), which suggests correct folding and structural integrity. Unfortunately, extensive efforts to obtain diffracting crystals for experimental structure analysis by X-ray crystallography of these two constructs (Section 2.8) proved unsuccessful. Thus, the energy-minimized AlphaFold model corresponding to expression construct CPL1_1-4 (Q20-T581) is shown in Supplementary Figure S3.

### 3.5. Activation and Proteolytic Activity of CPL1

**Figure 4:**
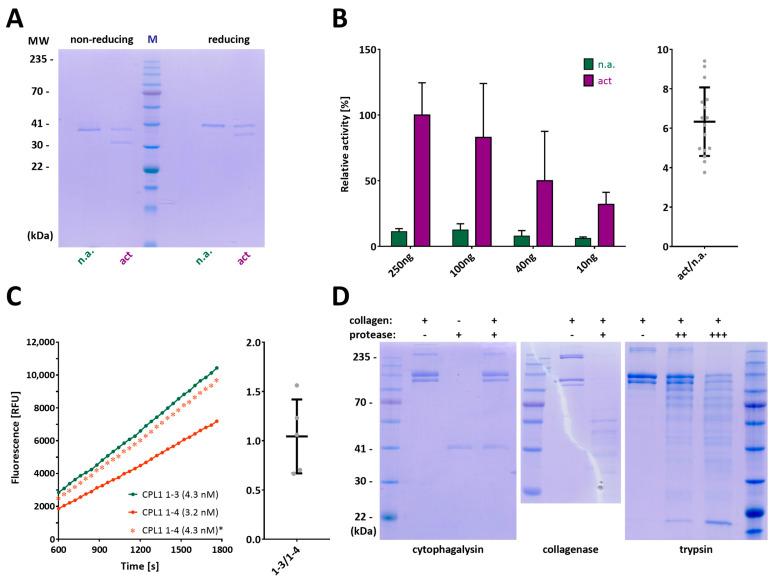
Trypsin-mediated activation and activity of CPL1 constructs. (**A**) SDS-PAGE analysis under non-reducing and reducing conditions, which shows that the trypsin-activated protease sample (act) shows a band ~7 kDa smaller than the non-activated sample (n.a.), which corresponds to the excision of the zymogenic N-terminal prosegment. (**B**) (**Left**) Average and standard deviation of relative activity of different amounts of activated wild-type CPL1_1-3 against the fluorogenic substrate DQ Gelatin (2 μg) compared to the non-activated zymogen. (**Right**) Ratio of activities between both protein variants (n = 16). (**C**) (**Left**) Fluorescence resulting from the turnover of DQ Gelatin by activated wild-type CPL1_1-3 and CPL1_1-4. The values shown in salmon for the latter are recalculated from the recorded curve at 3.2 nM and correspond to the same concentration as those for CPL1_1-3, and are therefore marked with an asterisk (*). (**Right**) Normalized molarity values for the two constructs (n = 5), which reveal equivalent activity. (**D**) SDS-PAGE analysis of the incubation of human type-I atelocollagen (10 μg) with 2 μg of wild-type CPL1_1-3 (**left**), 0.5 μg of *Clostridium histolyticum* collagenase (centre), and 0.1 μg (++) or 1 μg (+++) of bovine trypsin (right). The + and − signs indicate the presence or absence of collagen and the respective protease (cytophagalysin, collagenase, or trypsin, as labeled below the gels), with increasing + signs denoting higher protease concentrations. SDS-PAGE gels were cropped for clarity. For full gel images, please refer to Extended Data Figures S11 and S12 in the supplementary materials.

Given that the protein sequences included a predicted PS, which in MPs normally needs selective removal to yield the mature active CD [9], we assayed two enzymes commonly employed in limited proteolysis: trypsin and chymotrypsin. While the latter just led to indiscriminate degradation of the zymogenic CPL1_1-3 and CPL1_1-4 constructs, a clear cut was performed by trypsin, likely at bond K^64^–M^65^, which yielded a species ~7 kDa smaller than the precursor, as shown for CPL1_1-3 (Figure 4A). This would be compatible with the removal of the predicted PS (Q^20^–A^66^).

Next, we set out to test the activity of CPL1_1-3 and CPL1_1-4 through three series of experiments based on disparate biophysical approaches: a fluorogenic cleavage assay in solution with a fluorophore-labelled gelatin derivative (DQ Gelatin), gelatin zymography, and assessment through sodium dodecylsulfate polyacrylamide gel electrophoresis (SDS-PAGE) of the cleavage in solution of native type-I collagens from human and rat as substrates.

Activated CPL1_1-3 efficiently cleaved DQ Gelatin in a concentration-dependent manner, which indicates the protein is functional and well folded. Remarkably, the zymogen evinced a residual activity of up to one-sixth of that of the active form (Figure 4B,C), which points to the PS not binding, and thus inhibiting the CD very strongly under the present experimental conditions. Residual activity has been reported for other MP zymogens previously [9]. Activated CPL1_1-4 proved as active as CPL1_1-3 against this substrate (Figure 4C), in a concentration-dependent manner. In contrast and contrary to previous reports with the purified enzyme [34], the two constructs were inactive against native type-I collagens (see Figure 4D for human atelocollagen), which were efficiently degraded by a true bacterial collagenase [91,92] and, partially, by trypsin. Given that the latter serine peptidase does not degrade truly native type-I collagen [93], this result points to a certain degree of denaturation of the commercial collagen preparations employed. This suggests two possibilities: either one of the C-terminal cytophagalysin domains, which are absent in our constructs, is crucial for collagenolysis (similar to the role of the N-terminal activator domain in clostridial collagenases [91]), or this enzyme is misclassified as a true collagenase and should instead be considered a gelatinase. Unfortunately, our study cannot resolve this issue, as we were unable to obtain recombinant full-length protein.

**Figure 5:**
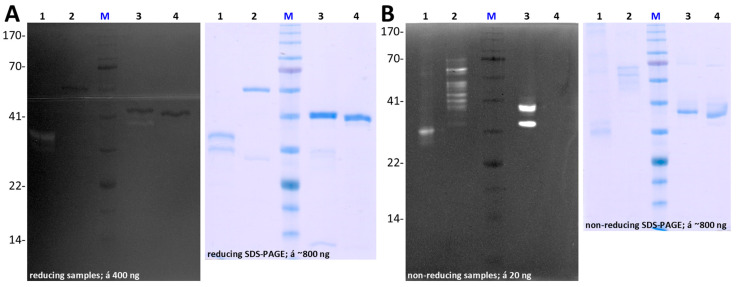
CPL1 activity in gelatin zymography. (**A**) Representative gelatin zymograms (**left**) and SDS-PAGE analysis (**right**) under reducing conditions of wild-type variants CPL1_1-2 (*lane 1*), CPL1_1-4 (*lane 2*), and CPL1_1-3 (*lane 3*), which evince only minute activity due to the unfolding of the protein variants caused by the reducing conditions, as well as of inactive CPL1_1-3 E^232^A-mutant (*lane 4*). (**B**) Same as (**A**) under non-reducing conditions, which locally preserves the integrity of the recombinant proteins, thereby aiding in-gel refolding and consequently revealing significant activity for wild-type CPL1_1-2 (*lane 1*), CPL1_1-4 (*lane 2*), and CPL1_1-3 (*lane 3*), but not for the mutationally inactivated CPL1_1-3 variant (*lane 4*). SDS-PAGE gels and zymograms were cropped for clarity. For full gel and zymogram images, please refer to Extended Data Figures S13 and S14 in the supplement.

Next, zymography employing gels imbedded with gelatin revealed the latter was efficiently degraded by CPL1_1-2, CPL1_1-3, and CPL1_1-4 (Figure 5A,B), which confirms previous findings [33]. This activity was ablated by introducing the E^232^A-mutation that removes the catalytic base/acid (Figure 5A,B), which provides evidence for our recombinant CPL1 constructs acting as bona fide MPs.

**Figure 6:**
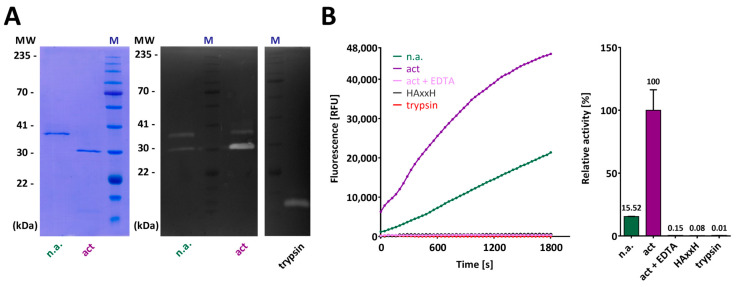
Inhibition of activity of CPL1. (**A**) (**Left**) Complete activation of CPL1_1-3 (*lane act*) by trypsin in SDS-PAGE, as shown by the absence of the zymogen band that is found in *lane n.a.* In zymography (**Centre**), both samples exhibited activity for both activated and non-activated CPL1, with significantly increased activity observed in the activated sample. Importantly, no trypsin activity was detected in the CPL1 samples. For reference, trypsin activity (Right) at an apparent molecular weight of ~18 kDa is shown. In zymography however, both samples demonstrated activity for both activated and non-activated CPL1 (**Centre**), with activity enriched in the activated sample. Note, no trypsin activity was observed in CPL1 samples, and trypsin activity is shown at an apparent molecular weight of ~18 kDa (**Right**). (**B**) The activity of CPL1_1-3 against fluorogenic DQ Gelatin is efficiently inhibited by EDTA as expected for a metallopeptidase, yielding only residual values that are comparable to those of the E^232^A-mutant and trypsin, which does not cleave this substrate. Note that the CPL1_1-3 zymogen still has a residual activity of ~15% of the active form. SDS-PAGE gels and zymograms were cropped for clarity. For full gel and zymogram images, please refer to Extended Data Figure S15 in the supplementary materials.

Finally, we further backed the latter by assaying ethylenediaminetetraacetate (EDTA), which is a potent chelator of divalent metals and thus acts as a non-specific inhibitor of MPs [94,95]. Indeed, activated CPL1_1-3, which was active in gelatin zymography (Figure 6A), was inhibited in the fluorogenic assay against DQ Gelatin to a comparable level as the E^232^A-mutation (Figure 6B), in agreement with previous findings [34]. As expected, trypsin was completely inactive due to the presence of the broad-spectrum serine peptidase inhibitor 4-(2-aminoethyl)benzenesulfonyl fluoride (AEBSF) in the assay conditions, effectively eliminating any trypsin activity after CPL1 activation (Figure 6B).

## 4. Conclusions

CPL1 is a multidomain member of the LOPAP subfamily of the pappalysins, which belong to the metzincin clan of MPs. In addition to a PS and a metzincin-type CD strongly reminiscent of ulilysin, computational predictions of the full-length 137 kDa protein hypothesize that it comprises eight Ig-like domains (D3–D10) arranged as three intercalated pairs of highly similar architecture (D3/D5, D4/D6, and D7/D9) plus two unique domains, D8 and D10. The latter could potentially play the role of the CTD described for T9SSs, one of which has been described for Cytophaga spp. Based on this structure prediction, our recombinant protein expression and purification studies enabled us to produce functional constructs spanning up to four domains (PS+CD+D3+D4), which are active against gelatin in two different experimental setups. These studies further confirmed that our recombinant CPL1 is an MP that is inhibited by mutation of its catalytic glutamate and by the broad-spectrum chelator, EDTA.

## Data Availability

All data are provided within the manuscript or supplementary information files.

## Supplementary Materials

The following supporting information can be downloaded at: https://www.mdpi.com/article/10.3390/biom14121604/s1, Figure S1: Protein expression and purification of full-length cytophagalysin; Figure S2: Side-by-side comparison of reducing and non-reducing SDS-PAGE conditions; Figure S3: AlphaFold model of expression construct CPL1_1-4; Figure S4: DNA sequence of the various tested cytophagalysin variants; Figure S5: Amino acid sequence of the various tested cytophagalysin variants; Figure S6: PCR and mutagenesis primers used in this study; Table S1: Structural validation statistics of the full-length cytophagalysin homology model; Extended Data Figures S7–S21: Non-cropped SDS-PAGE and zymogram images used for figure preparation. PDB file of the geometry-optimized cytophagalysin (UniProt ID: Q46348, residues Q20–K1298) AlphaFold2 model utilized for structural analysis.

## Abbreviations

ADAMs	adamalysins and a-disintegrin-and-metallopeptidase enzymes
AEBSF	4-(2-aminoethyl)benzenesulfonyl fluoride
CD	catalytic domain
CPL1	cythophagalysin from Cytophaga strain L43-1
CSD	C-terminal subdomain
EDTA	ethylenediaminetetraacetate
FNIII	fibronectin type-III domain
Ig	immunoglobulin
Ig-like	immunoglobulin-like
IPTG	isopropyl-β-D-1-thiogalactopyranoside
LNKs	linkers
LOPAPs	lower pappalysins
MP	metallopeptidase
NSD	N-terminal subdomain
PDB	Protein Data Bank
pLDDT	predicted local-distance difference test
PS	prosegment
SDS-PAGE	sodium dodecylsulfate polyacrylamide gel electrophoresis
SEC	size-exclusion chromatography
SP	signal peptide
T9SS	type-IX secretion system
TEV	tobacco-etch virus peptidase
TLCK	N-tosyl-L-lysinyl chloromethyl ketone
TPCK	N-tosyl-L-phenylalanyl chloromethyl ketone
UP	UniProt database
