# Supplementary material for: Dissecting Cytophagalysin: Structural and Biochemical Studies of a Bacterial Pappalysin-Family Metallopeptidase
Source: Biomolecules. 2024 Dec 16;14(12):1604. doi: 10.3390/biom14121604 (PMC11674741; doi:10.3390/biom14121604)
Supplement: Supplementary file 1 [file biomolecules-14-01604-s001.zip › Estevan-Morio_Biomolecules-2024_Supplement.pdf]

Supplementary Information

Dissecting Cytophagalysin: Structural and Biochemical Studies of a Bacterial Pappalysin-Family Metallopeptidase

Eva Estevan-Morió, Juan Sebastián Ramírez-Larrota, Enkela Buschi, and Ulrich Eckhard *

Synthetic Structural Biology Group, Molecular Biology Institute of Barcelona (IBMB), Spanish National Research Council (CSIC), 08028 Barcelona, Spain
* Corresponding: ulrich.eckhard@ibmb.csic.es

Supplementary Figures:

Supplementary Figure S1: Protein expression and purification of full-length cytophagalysin.

Supplementary Figure S2: Side-by-side comparison of reducing and non-reducing SDS-PAGE conditions.

Supplementary Figure S3: AlphaFold model of expression construct CPL1_1-4.

Supplementary Figure S4: DNA sequence of the various tested cytophagalysin variants.

Supplementary Figure S5: Amino acid sequence of the various tested cytophagalysin variants.

Supplementary Figure S6: PCR and mutagenesis primers used in this study.

Supplementary Tables:

Supplementary Table 1: Structural validation statistics of the full-length cytophagalysin homology model.

Extended Data Figures S7-S21:

Non-cropped SDS-PAGE and zymogram images used for figure preparation.

Supplementary Figures

Supplementary Figure S1.

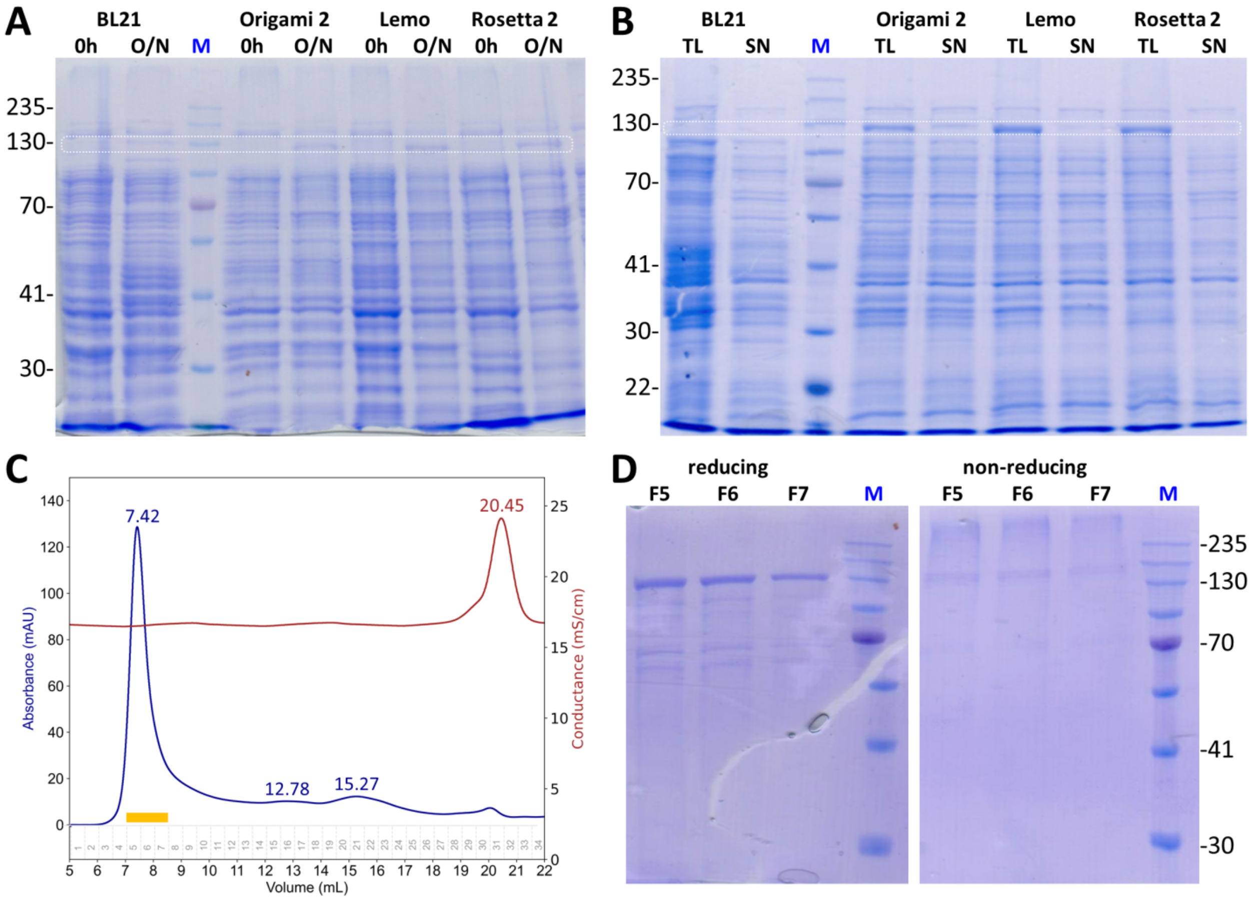

Supplementary Figure S1. (A) Protein expression screening of full-length wild-type CPL1 (Q[20]–K[1282]) cloned into vector pCri7a* in *Escherichia coli* strains BL21(DE3), Origami 2(DE3), Lemo21(DE3) and Rosetta(DE3). A faint expression band at the expected molecular weight (139 kDa) was observed (framed) in all strains after SDS-PAGE analysis and staining, with BL21(DE3) giving the lowest yield. **(B)** Protein solubility assessment by comparison of the total lysate (TL) with the soluble fraction after clarification of the supernatant (SN) through centrifugation. Most of the target protein was insoluble. Origami 2(DE3) was identified as the best host for soluble CPL1 expression. **(C)** Size-exclusion chromatography purification of full-length CPL1 expressed in Origami 2(DE3) cells, after previous nickel-affinity purification. The protein is mostly eluted in the void peak at 7.42 mL, which points to the presence of soluble aggregates or partially unfolded protein. **(D)** SDS-PAGE analysis at both reducing and non-reducing conditions of the contents of the peak shown in (C). While the reducing gel shows a predominant band at the expected molecular weight, the non-reducing gel reveals the presence of inter-molecular disulfide bridges, which indicate the presence of a non-native disulfide network and improper protein folding. SDS-PAGE gels were cropped for clarity. For full gel images, please refer to Extended Data Figures S16-S18 further below in the supplement.

Supplementary Figure S2.

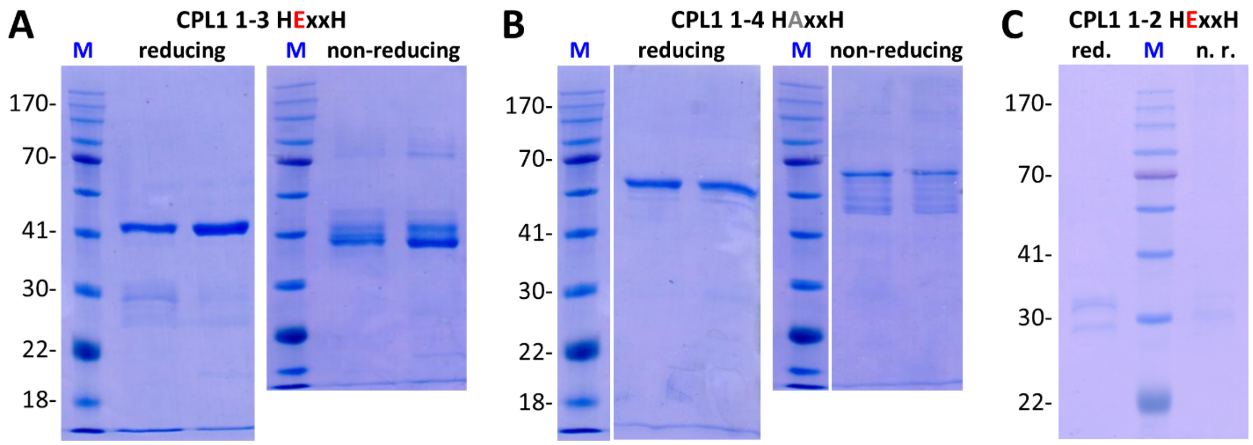

Supplementary Figure S2. Representative SDS-PAGE analysis under reducing (left) and non-reducing (right) conditions of the C-terminally deletion constructs **(A)** wild-type CPL1_1-3 (~47 kDa); **(B)** CPL1_1-4 mutant $E^{232}A$ (~63 kDa); and **(C)** wild-type CPL1_1-2 (~35 kDa). Importantly, all samples exhibited distinct bands at their expected molecular weight under reducing conditions. However, multiple bands were observed when using non-reducing SDS-PAGE sample buffer, which indicates inconsistent formation of disulfide bridges and suggests the presence of incomplete or partially incorrect disulfide networks in the samples. SDS-PAGE gels were cropped for clarity. For full gel images, please refer to Extended Data Figures 19-21 further below in the supplement.

Supplementary Figure S3.

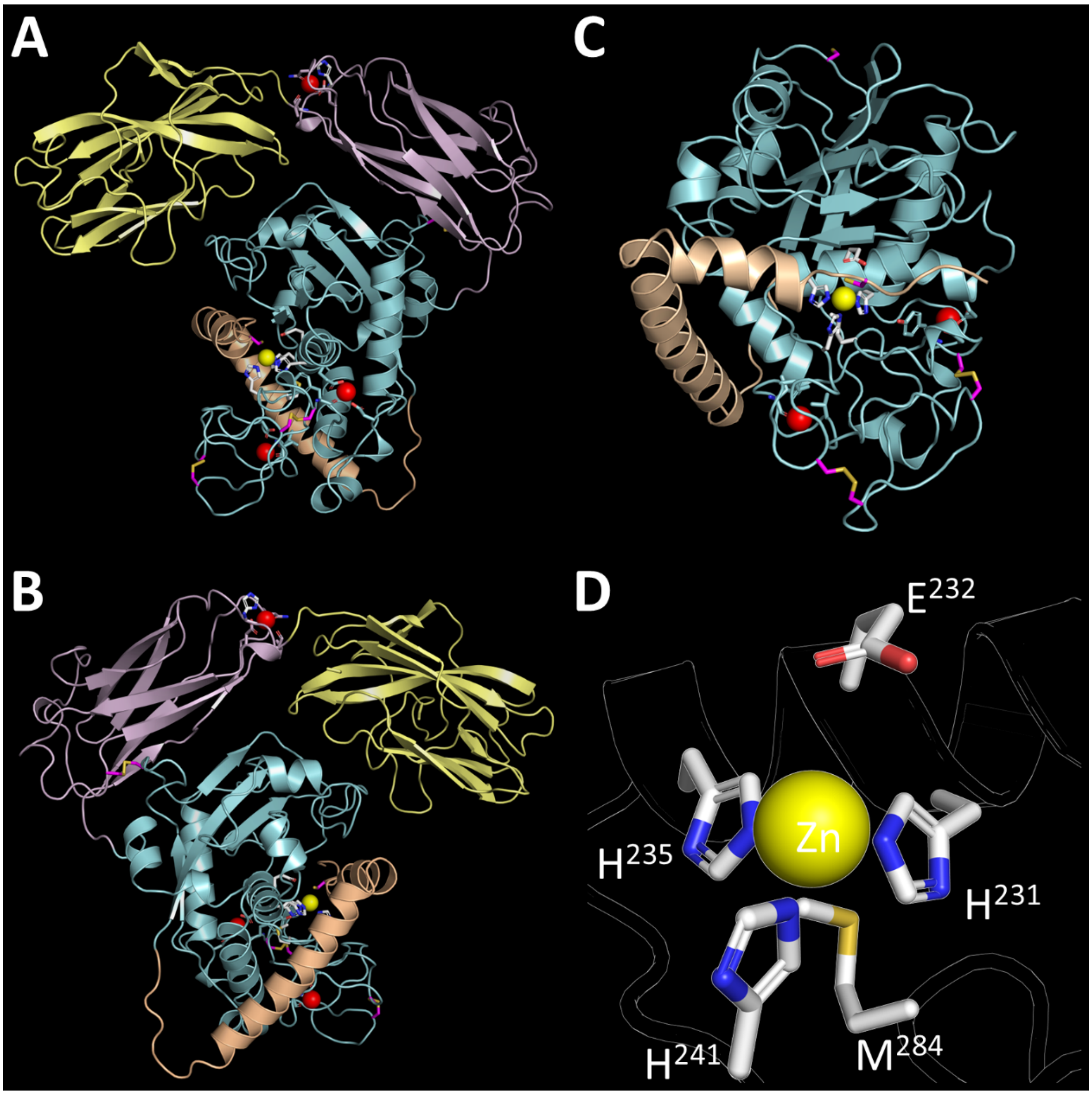

Supplementary Figure S3. AlphaFold model of expression construct CPL1_1-4 (Q20-T591) with subdomains color-coded as in Figure 1: prodomain in light salmon, catalytic domain in crystal blue, and immunoglobulin-like domains D3 and D4 in light orchid and yellow, respectively. Side views of CPL1_1-4 are shown in **(A)** and **(B)**, while a front view of prosegment and catalytic domain alone is shown in **(C)**. Potential disulfide bridges and free cysteines are depicted in magenta, and the active site zinc ion is displayed as a yellow sphere. Putatively bound calcium ions are highlighted in red. (D) Close-up view to the active site. Three histidine residues H^{231}, H^{235}, H^{241}) coordinate the active site zinc ion, with M^{284} further stabilizing the zinc by providing a hydrophobic base. Glu^{232} functions as a general base/acid during catalysis, acting through a water molecule bound between its side chain oxygen and the zinc.

Supplementary Figure S4.

cc**ATG**gctagctggagccacccgcagttcgagaaaggcagtggttctgagaatctttattttcagggccat**ATG**	
**CAA**ggactaccctgtaggacatcagaagagaacgagaagatctacaagagcaacccgcatgcattgaaggaaaagcagga<br>tttcgacaccttctcaaaaaacttttccgctgcccgtaaaagcaaaaccggcaagatggctgagacctcttatgttatcc<br>cggtagtgttccacatttacggcgatgttcagagcggtaagaccgttacgtatcagaaaatcgtgaatcatctgcaacat<br>ctgaatgatgattttaacggccgtaatgctgactaccagaccgtcgatccgtttttccaagcacgtagaggtacgttgaa<br>gatcgagttcaaactcgcgaaaatcgacccgaacggcggttgtacttcaggtgttgtctttcacccggcgaaaaatggtt<br>tcggtaacggcggcggctacgacgaccagatcgcggcggacgcatgggataataccaaatacatgaatgtgtatatccag<br>aatgacctatacgccgacggtgcgttgaacaacagcggtgtggcgtggtatccggatagcggcatgaccgcgtcgaacac<br>cgcgcgcgtggtgtttaatggcgcctacctgtatgacaacagctattctactgagttctccgcgaccctaacc**cacgaa**t<br>ttggt**cat**ttctgaacctgatt**cat**accttcgagggtggttgcaccggcactgacgaagttgatgacaccccgttagag<br>gatggcaagcacaccctggcatgtacccaaggcaccaactgcaatggcgaccgcgttaataacgagaactacatgggcta<br>caatggtgctcaaggttgctacaagatgtatacccaaggtcagattgatcgtatggttgccgctctggaacaccctagcc<br>gcaaatcgctttggcaggctgcgaacctgattgcgaccggtgtt**AAC**	**Q20-**<br><br><br><br><br><br><br><br><br><br><br>**-N328**
agcacaggtggtctactcactgtcaacacgagctcgttcaaagaagcaatttcaaataacggcagcttcgataccgcgag<br>catcgttacgctgtccggcaagacgtttgcactgagctcgggcaccctcaccagcggtactcactttagccacaccttcc<br>cggcgggcatcaccccctgtcattactgttaactccaattctcagcttaccattaccttgacgggcaatgccacgagccac<br>cagaccgtgaacaacgccaagggtgcgattacgttcctgccagcagcgtttaccggcggcaccgtggacctgacgtgcac<br>ctccctgaattatgactttaagttc**GTC**	 <br><br><br>**-V444**
gacccgtacggtatcttctttgttgacatgccggatgcaacagcttcggctgctcaaacctggaaagcatttaagatcga<br>caaaggtgatgacccggctttcggtagctggcgttataccgcgaatgccctgaagatcgagacgtacgcgaaaaaactgg<br>tatgtgaaaccggcacccgtaacatcacattgctgccggcaaacagccccatcagtgcgtcctcaaactttacggctccg<br>ggtgcgtacccgaaccaactggatttgcgtaccgcgtcttacaccaattgggatggaaagaccggctatgtgggattcga<br>ttacctgattgacggcttgacgtgctacggttggtttaaagtgaccgtggatacgaacggtgacggcttctcgataaccg<br>agtatgcgtacaacacccaaccgggtgcgaccatttac**ACG**	 <br><br><br><br>**-T591**
ggcatgaccccgaaaacggccgttgtcctgtccaccgacactttatatgaggcggacgctaatgacgggggtattacatc<br>taatacggtcatcagcttgtccaccaataacggcactttcaccaaatctaccggcacgttcaccgcgaataccgactata<br>ccatcaccggggtgccggcgggtctgacagcggttcttactttacaatcgaacagcaaagccgcgctgacgtttacgggc<br>aaggccagctctcacctgccagcgaacgacgccgcggtgattgtgaccttcaaggatgcagcaattaccggtggcattac<br>gactctggacaacgcagctaagacgattaatctgaagtttgagagcccgtatggtatttattatgttaacaatccggatt<br>atattgcatccccggccgcggtgtggcaatattttgatctgggtattggcgataacaccgaatacggtgcctggcagtac<br>gcagcaaacgcgctcaagatcgaaacctatggcaagcgtctggtcaccgagacgggtactcgcaacattaccaaaattgc<br>ggcgggtgcgtccatcggcgccacctctaacttcaccgcgccgggcgcctatccgaatcagctggatctgcgcacggcga<br>gctacaccgcgtgggacaaccagaccgcctacgtcggtttcgagtatagcagacgtggtcgaacatgctacggctggttc<br>aaggtgaaagttaccgcgaacggtgacggttacaccatcagcgagttcgcctacaatacacaaccgaaccaggccatcat<br>cgcaggtgctaccggcggcattactgtcgcggcgccgacgaacctggtggctaccgcaaattccaccagcttgcaagcgg<br>tgctgacttgggttgacaacgcgacgaacgaaaccggttacaccgtcgagcgtgcaagcgcagatggcgtttatacctcg<br>atcgccacacttggcgcgaacgtggtcacctatacgaataccggcttgacagcgggcaacacctacacctaccgcgtgaa<br>ggcgaacgccaacgccgttagcagcgactattcgaacgtggcgaccgcggtgatcgaagcagcgagtgcctactgcaccg<br>cccagggtagcaacagttatgagtacatcaaagatgttaccatcggctcgttcaccaccacctctggcgcggacgcatcc<br>ggttatggtaactacacctccaagaatattaccttgaccccaaatacgtctgctgcggtcagcctgactccaggtttcag<br>cggtagcaactatagcgaaagctggggtatctggatcgattataacaagaacaacaccttcgagccgagcgaaaaagtta<br>ttaatggtctgtcttctaccggcactgtaacgggtaactttaccgttgccgacggcaccggtactacccgcatgcgtatt<br>gttatgaaatacaatggtaacccggcgaccccgtgtgataacatcggcgacggtgaagttgaagactacaccgtaacctt<br>cggcgcatccaatccgaatccagttaccttgaacactccgaccaacattggcaattcgggtgtttattcttccggtttt<br>atgcgagctggaccgaggttgccactgctaccagctacgaagtgcagctgtacaaagctgcgaccggttggaccaccacg<br>ggcaccagcaccacctactacctgtggattccgaaacagggcaccgagactgcgtaccgttttcgtgtgcgtgcagcgaa<br>cgcgtctggcaccagcgcttggagcaactatctggatattaacctgccgctgtccggtgctgaaggtaatgacttactta<br>tgggtaagaagagctttagcatgtttccgaatccagccactgacgttgtgaattttaacttcgaaaacgtgaacatcccg<br>acagcagtgatcaccatttacaacaacgtcggtgctgtaatcgataccgtgcgcaacacatccagctacacgctgaagaa<br>cgttccgaccggcatctaccatgttgttctgactgatggcaattttacccaacgtaaaaaactgatcgtt**AAA**	 <br><br><br><br><br><br><br><br><br><br><br><br><br><br><br><br><br><br><br><br><br><br><br><br>**-K1282**
ggatctggtcatcaccaccaccatcaccaccactaataaggatcc	

Supplementary Figure S4. Nucleotide sequence of full-length CPL1 (Q^{20}–K^{1282}) in the *E. coli* expression plasmid pCri7a*. Cloning sites are highlighted in yellow, with *Nco*I and *Nde*I before and after the TEV-cleavable Strep-tag, respectively, and *Bam*HI at the 3'-end right of the non-cleavable His_8-tag. Amino-acid boundaries of the tested constructs are indicated on the right (CPL1_1-2: Q^{20}–N^{328}; CPL1_1-3: Q^{20}–V^{444}; CPL1_1-4: Q^{20}–T^{591}), and the codons for the zinc-binding histidine residues of the active site HExxHxxGxxH motif are highlighted in blue, while that of the general base glutamate (E^{232}) is indicated in red. This residue is mutated to alanine in the inactivated E^{232}A construct variants through codon *gcc*. Of note, for constructs lacking the N-terminal Strep-tag, the nucleotide sequences were introduced into the *E. coli* expression plasmid pCri7b using *Nde*I and *Bam*HI restriction sites.

Supplementary Figure S5.

MASWSHPQFEKGSGSENLYFQGHM	
**Q**GLPCRTSEENEKIYKSNPHALKEKQDFDTFSKNFSAARKSKTGKMAETSYVIPVVFHIYGDVQSGKTVTYQ<br>KIVNHLQHLNDDFNGRNADYQTVDPFFQARRGTLKIEFKLAKIDPNGGCTSGVVFHPAKNGFGNGGGYDDQI<br>AADAWDNTKYMNVYIQNDLYADGALNNSGVAWYPDSGMTASNTARVVFNGAYLYDNSYSTEFSATLT**HE**FG**H**<br>FLNLI**H**TFEGGCTGTDEVDDTPLEDGKHTLACTQGTNCNGDRVNNENYMGYNGAQGCYKMYTQGQIDRMVAA<br>LEHPSRKSLWQAANLIATGV**N**	**Q20-**<br><br><br><br>**-N328**
STGGLLTVNTSSFKEAISNNGSFDTASIVTLSGKTFALSSGTLTSGTHFSHTFPAGITPVITVNSNSQLTIT<br>LTGNATSHQTVNNAKGAITFLPAAFTGGTVDLTCTSLNYDFKF**V**	 **-V444**
DPYGIFFVDMPDATASAAQTWKAFKIDKGDDPAFGSWRYTANALKIETYAKKLVCETGTRNITLLPANSPIS<br>ASSNFTAPGAYPNQLDLRTASYTNWDGKTGYVGFDYLIDGLTCYGWFKVTVDTNGDGFSITEYAYNTQPGAT<br>IY**T**	 <br>**-T591**
GMTPKTAVVLSTDTLYEADANDGGITSNTVISLSTNNGTFTKSTGTFTANTDYTITGVPAGLTAVLTLQSNS<br>KAALTFTGKASSHLPANDAAVIVTFKDAAITGGITTLDNAAKTINLKFESPYGIYYVNNPDYIASPAAVWQY<br>FDLGIGDNTEYGAWQYAANALKIETYGKRLVTETGTRNITKIAAGASIGATSNFTAPGAYPNQLDLRTASYT<br>AWDNQTAYVGFEYSRRGRTCYGWFKVKVTANGDGYTISEFAYNTQPNQAIIAGATGGITVAAPTNLVATANS<br>TSLQAVLTWVDNATNETGYTVERASADGVYTSIATLGANVVTYTNTGLTAGNTYTYRVKANANAVSSDYSNV<br>ATAVIEAASAYCTAQGSNSYEYIKDVTIGSFTTTSGADASGYGNYTSKNITLTPNTSAAVSLTPGFSGSNYS<br>ESWGIWIDYNKNNTFEPSEKVINGLSSTGTVTGNFTVADGTGTTRMRIVMKYNGNPATPCDNIGDGEVEDYT<br>VTFGASNPNPVTLNTPTNIGNSGVYSSGFYASWTEVATATSYEVQLYKAATGWTTTGTSTTYYLWIPKQGTE<br>TAYRFRVRAANASGTSAWSNYLDINLPLSGAEGNDLLMGKKSFSMFPNPATDVVNFNFENVNIPTAVITIYN<br>NVGAVIDTVRNTSSYTLKNVPTGIYHVVLTDGNFTQRKKLIV**K**	 <br><br><br><br><br><br><br><br>**-K1282**
GSGHHHHHHHH**	

Supplementary Figure S5. Amino-acid sequence of full-length wild-type CPL1 (Q^{20}–K^{1282}) as expressed in *E. coli* from plasmid pCri7a*. The N-terminal TEV-cleavable Strep-tag, and the C-terminal non-cleavable His_8-tag are indicated, and the amino-acid boundaries of the various constructs are shown on the right (CPL1_1-2: Q^{20}–N^{328}; CPL1_1-3: Q^{20}–V^{444}; CPL1_1-4: Q^{20}–T^{591}). The zinc-binding histidine residues of the active site HExxHxxxxxH motif are highlighted in blue, while the general base glutamate (E^{232}) is indicated in red (and mutated to alanine in the inactivated $E^{232}A$ construct variants). After shuffling the respective constructs into plasmid pCri7b *via Nde*I and *Bam*HI sites, constructs started with MQ^{20}.

Supplementary Figure S6. A

Inverse PCR Cloning Primers

**CPL1-FWD**	5′-TCT GGT CAT CAC CAC CAC C-3′
**CPL1-REV_N328**	5′-GTT AAC ACC GGT CGC AAT CAG-3′
**CPL1-REV_V444**	5′-GAC GAA CTT AAA GTC ATA ATT CAG G-3′
**CPL1-REV_T591**	5′-CGT GTA AAT GGT CGC ACC C-3′

B

Inverse PCR Mutagenesis Primers

**CPL1-E232A_FWD**	5′-GCC TTT GGT CAT TTT CTG AAC CTG ATT C-3′
**CPL1-E232A_REV**	5′-GTG GGT TAG GGT CGC GG-3′

C

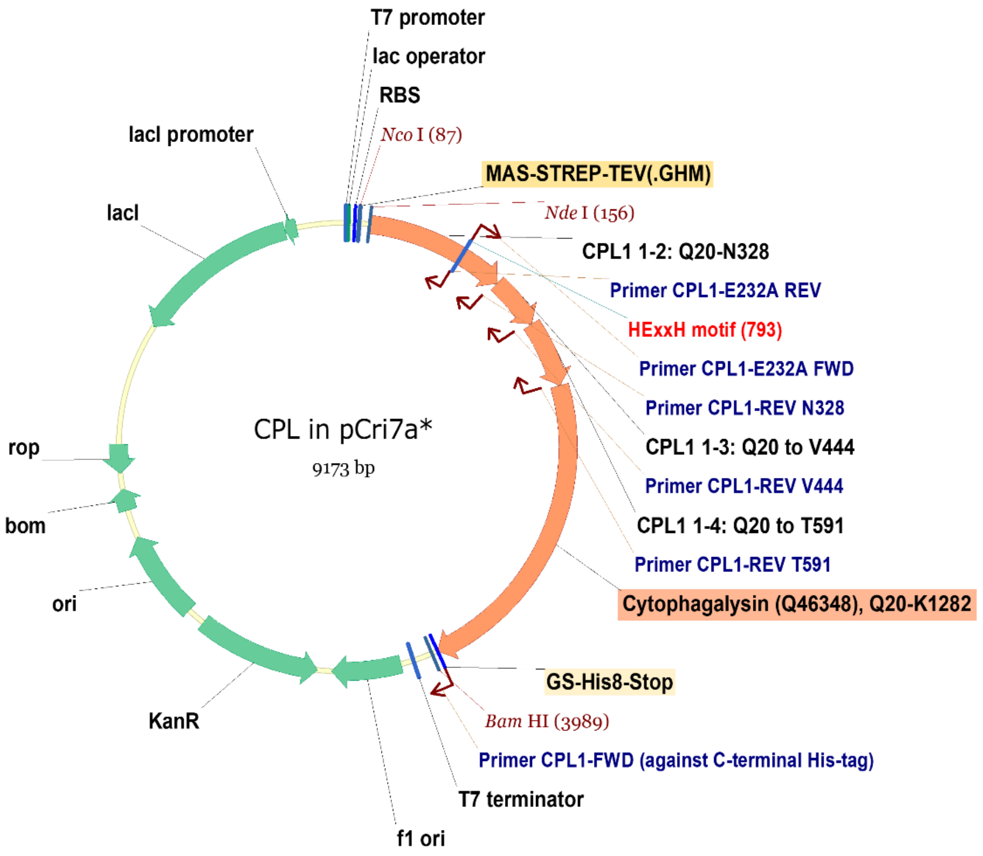

Supplementary Figure S6. (A) Primers used for inverse PCR amplification. Primer FWD is against the C-terminal His$_8$-tag. **(B)** Primers used for mutagenesis, the codon accounting for the E^{232}A mutation is highlighted in red. **(C)** Scheme of the pCri7a* expression plasmid to illustrate the primer binding sites.

Supplementary Tables

Supplementary Table S1. Structural validation statistics of the full-length cytophagalysin homology	
	CPL1
Total number of residues	1263
MolProbity Score	2.35
Clashscore	11.9
Ramachandran favoured	1227 (92.3%)
Ramachandran outliers	1 (0.08%)
Poor rotamers	69 (6.8%)
Favoured rotamers	812 (79.7%
Rama distribution Z-score	1.96
Cβ deviations (>0.25 Å)	0
Rmsd of bonds (in Å)	0.003
Bad bonds (out of total)	0 (9772)
Rmsd of angles (in °)	0.911
Bad angles (out of total)	5 (13,353)
Cis-prolines (out of total)	3 (44)
CaBLAM outliers	17
CA Geometry outliers	9
Chiral volume outliers	0
Statistics obtained with *Molprobity*[96]. The underlying geometry optimized structural model can be downloaded as a PDB file as part of the Supplementary Materials.	

Reference:

96. Williams, C. J. et al. MolProbity: More and better reference data for improved all-atom structure validation. Protein Sci. Publ. Protein Soc. 27, 293–315 (2018).

Non-cropped SDS-PAGE and Zymogram Images used for Figure Preparation

Extended Data Figure S7: non-cropped gel images of Figure 3A.

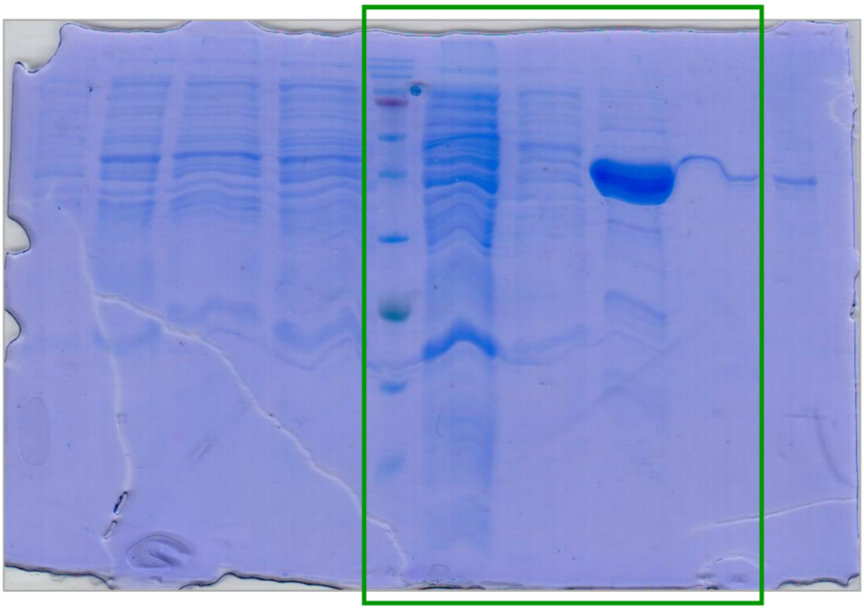

LANES 1-5

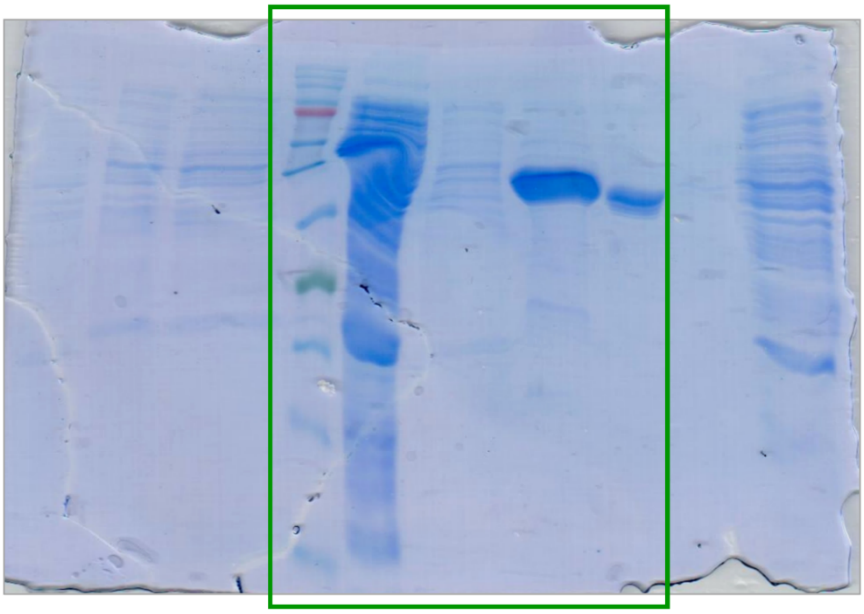

LANES 6-10

Extended Data Figure S7. Nickel-affinity purification of CPL1_1-3 (Q20–V444) in its wild-type (top panel; HExxH) and E232A-mutant (bottom panel; HAxxH) variants. The full, uncropped SDS-PAGE gels corresponding to **Figure 3A** are provided, with the cropped regions indicated by a red box for clarity.

Extended Data Figure S8: non-cropped gel images of Figure 3B.

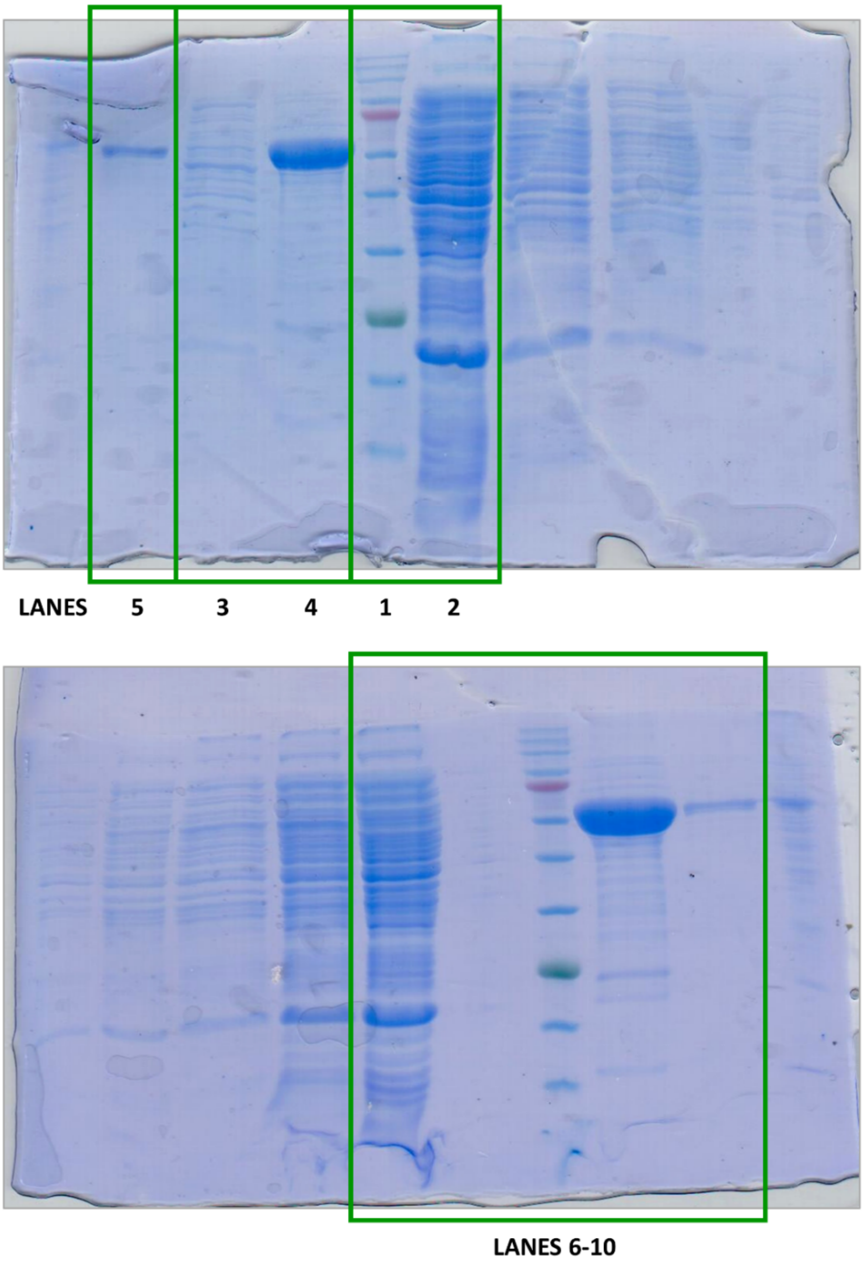

Extended Data Figure S8. Nickel-affinity purification of CPL1_1-4 (Q20–T591) in its wild-type (top panel; HExxH) and E232A-mutant (bottom panel; HAxxH) variants. The full, uncropped SDS-PAGE gels corresponding to **Figure 3B** are provided, with the cropped regions indicated by a red box for clarity. Note: When loading the gel, the samples for W20 and E250-1 were mistakenly loaded in the wrong order.

Extended Data Figure S9: non-cropped gel images of Figure 3D.

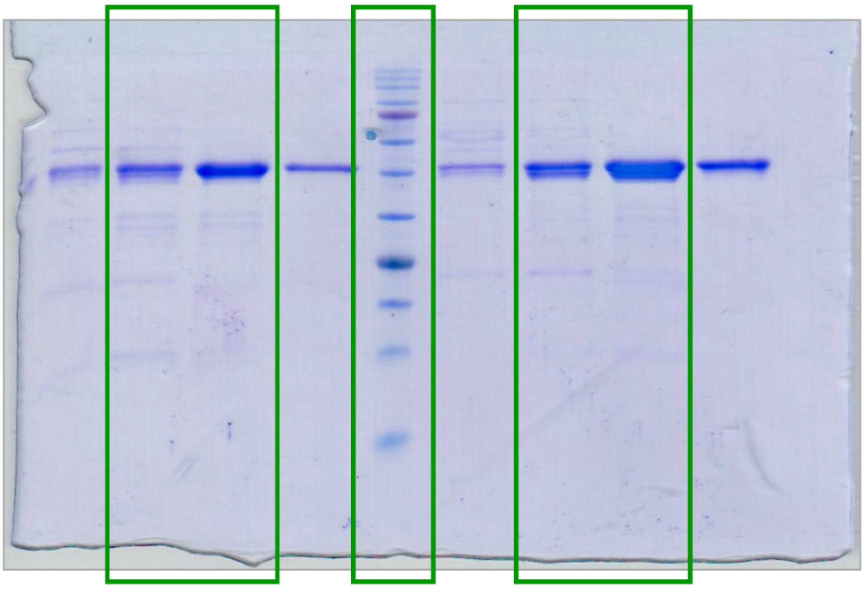

Extended Data Figure S9. SDS-PAGE analysis of the size exclusion chromatography analysis of CPL1_1-3 (Q20–V444) in its wild-type (lanes 2-3; panel; HExxH) and E232A-mutant (lanes 4-5; HAxxH) variants. The full, uncropped SDS-PAGE gel corresponding to **Figure 3D** is provided, with the cropped regions indicated by a red box for clarity.

Extended Data Figure S10: non-cropped gel images of Figure 3F.

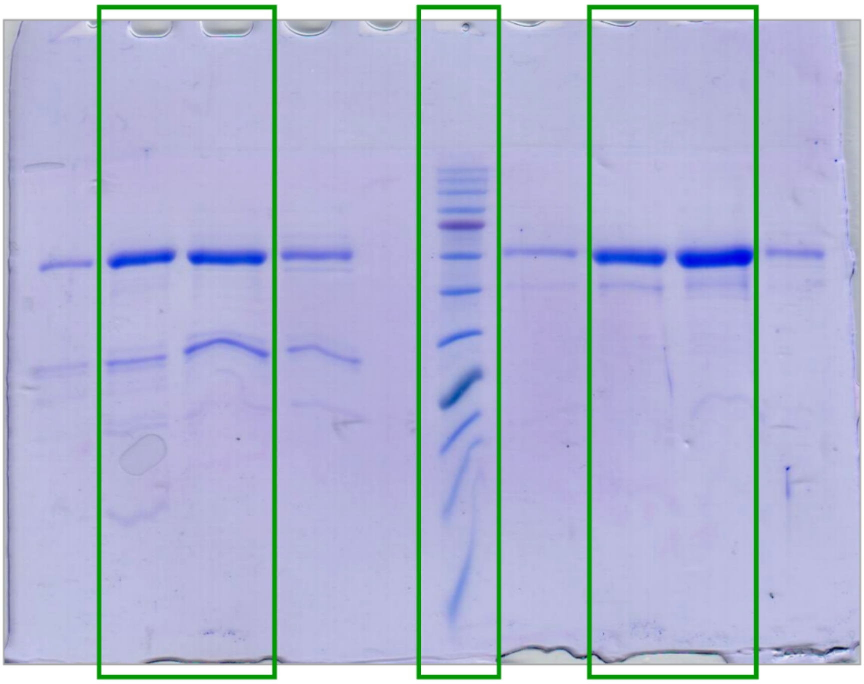

Extended Data Figure S10. SDS-PAGE analysis of the size exclusion chromatography analysis of CPL1_1-4 (Q20–T591) in its wild-type (lanes 2-3; panel; HExxH) and E232A-mutant (lanes 4-5; HAxxH) variants. The full, uncropped SDS-PAGE gel corresponding to **Figure 3F** is provided, with the cropped regions indicated by a red box for clarity.

Extended Data Figure S11: non-cropped gel images of Figure 4A.

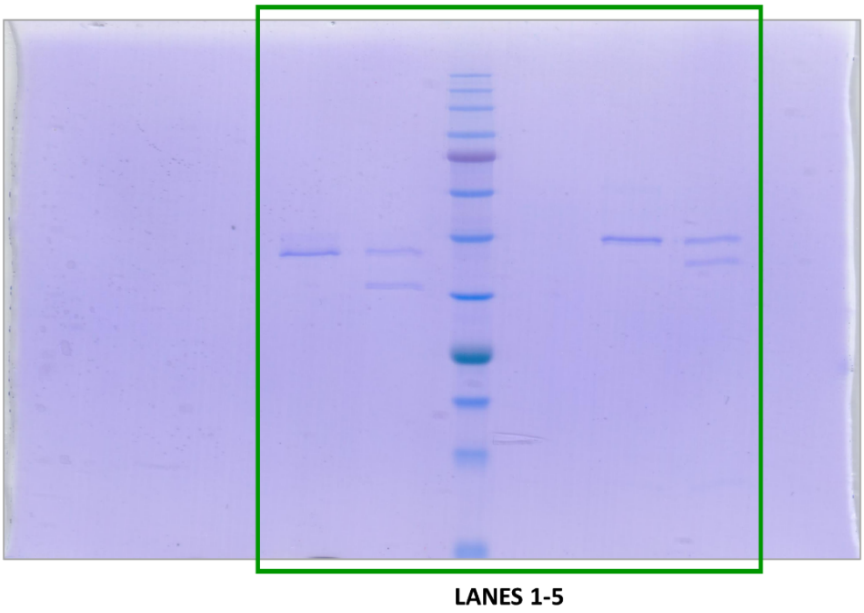

Extended Data Figure S11. SDS-PAGE analysis of trypsin-mediated CPL1_1-3 (Q20–V444) activation under non-reducing (left; lanes 1-2) and reducing conditions (right; lanes 4-5). The full, uncropped SDS-PAGE gel corresponding to **Figure 4A** is provided, with the cropped region indicated by a red box for clarity. Note: The lane immediately to the right of the molecular weight marker was not loaded.

Extended Data Figure S12: non-cropped gel images of Figure 4D.

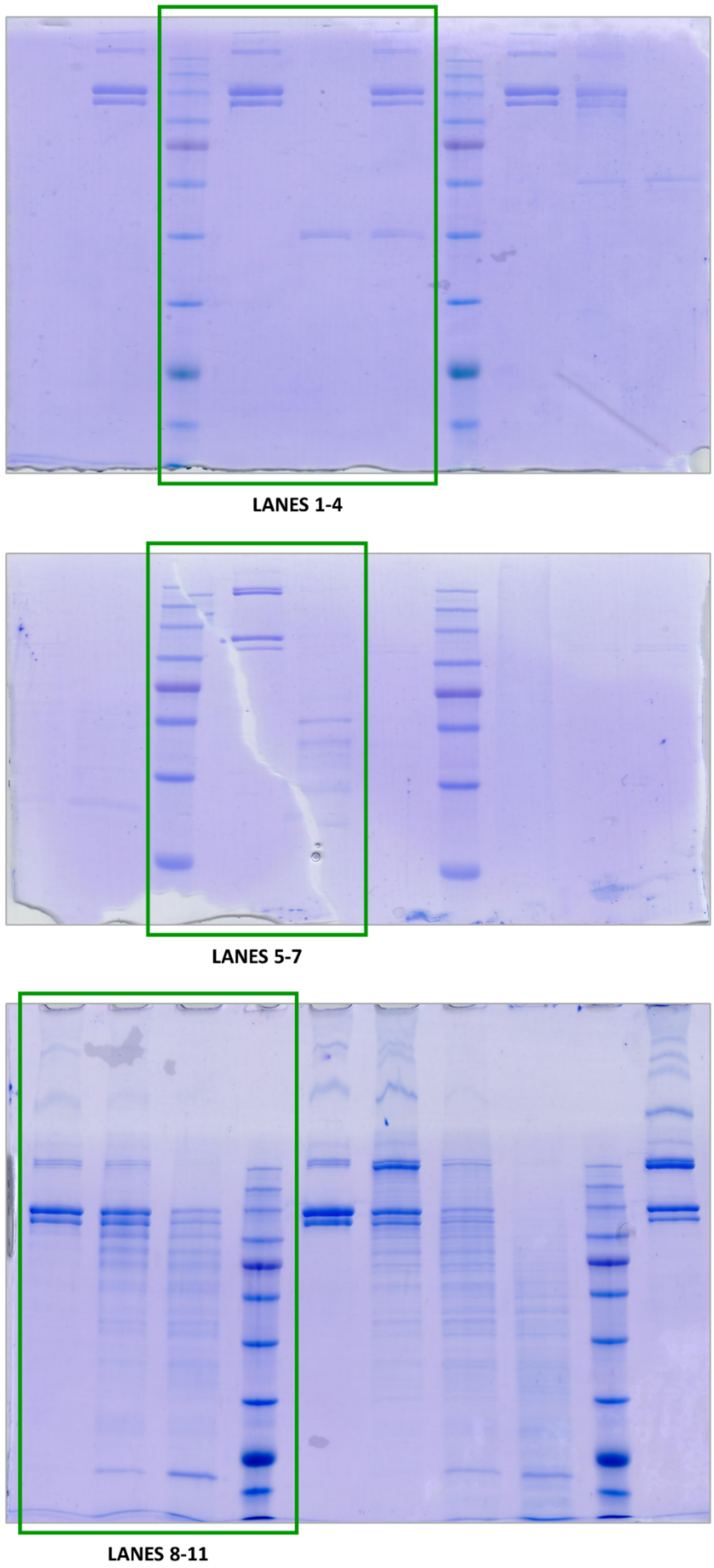

Extended Data Figure S12. SDS-PAGE analysis of the incubation of human type-I atelocollagen (10 µg) with 2 µg of wild-type CPL1_1-3 (top panel; lanes 1-4), 0.5 µg of Clostridium histolyticum collagenase (middle panel; lanes 5-7) and 0.1 µg or 1 µg of bovine trypsin (bottom panel; lanes 8-11). The full, uncropped SDS-PAGE gels corresponding to **Figure 4D** are provided, with the cropped regions indicated by a red box for clarity.

Extended Data Figure S13: non-cropped gel images of Figure 5A.

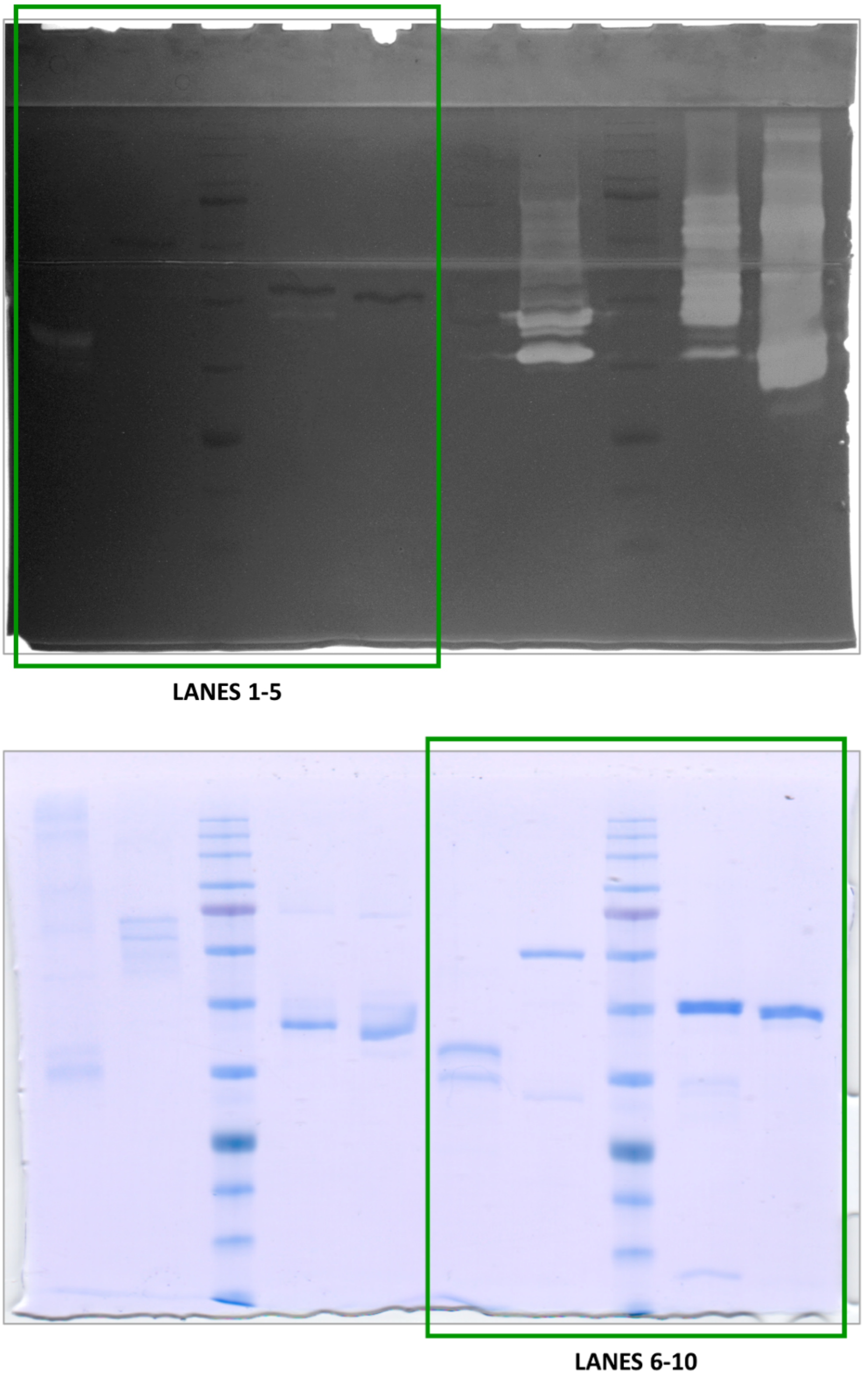

Extended Data Figure S13. Gelatin zymogram (top panel; lanes 1-5) under reducing conditions of wild-type variants CPL1_1-2 (lane 1), CPL1_1-4 (lane 2) and CPL1_1-3 (lane 4), as well as of inactive CPL1_1-3 E232A-mutant (lane 5). SDS-PAGE analysis (bottom panel; lanes 6-10) under reducing conditions of wild-type variants CPL1_1-2 (lane 6), CPL1_1-4 (lane 7) and CPL1_1-3 (lane 9), as well as of inactive CPL1_1-3 E232A-mutant (lane 10). The full, uncropped zymogram and SDS-PAGE gel corresponding to **Figure 5A** are provided, with the cropped regions indicated by a red box for clarity.

Extended Data Figure S14: non-cropped gel images of Figure 5B.

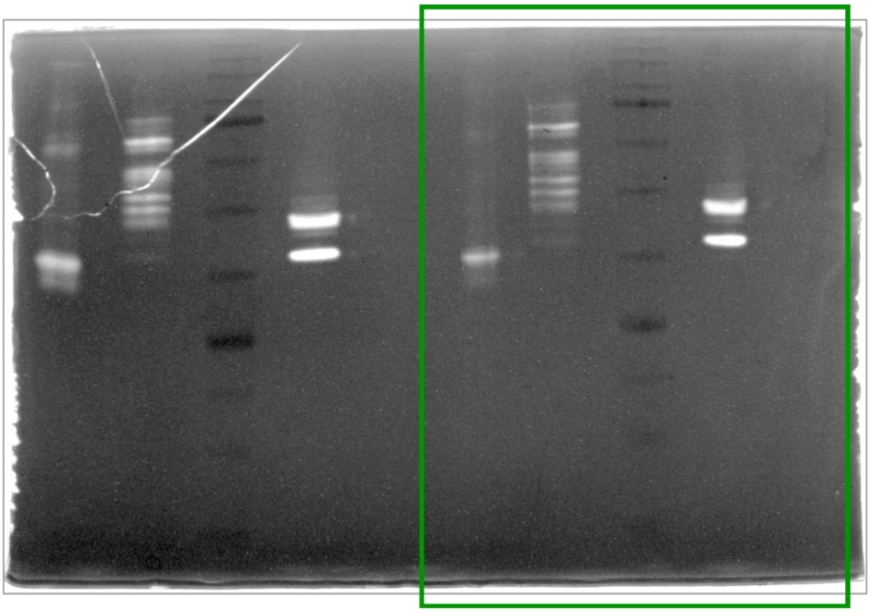

LANES 1-5

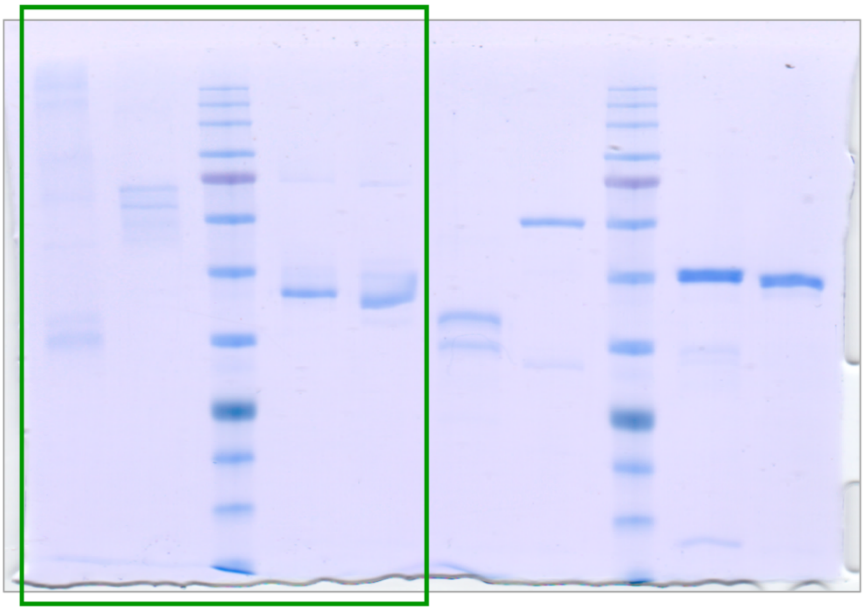

LANES 6-10

Same SDS-PAGE gel as used for Figure 5A.

Extended Data Figure S14. Gelatin zymogram (top panel; lanes 1-5) under <u>non-reducing conditions</u> of wild-type variants CPL1_1-2 (lane 1), CPL1_1-4 (lane 2) and CPL1_1-3 (lane 4), as well as of inactive CPL1_1-3 E232A-mutant (lane 5). SDS-PAGE analysis (bottom panel; lanes 6-10) under non-reducing conditions of wild-type variants CPL1_1-2 (lane 6), CPL1_1-4 (lane 7) and CPL1_1-3 (lane 9), as well as of inactive CPL1_1-3 E232A-mutant (lane 10). The full, uncropped zymogram and SDS-PAGE gel corresponding to **Figure 5B** are provided, with the cropped regions indicated by a red box for clarity.

Extended Data Figure S15: non-cropped gel images of Figure 6A.

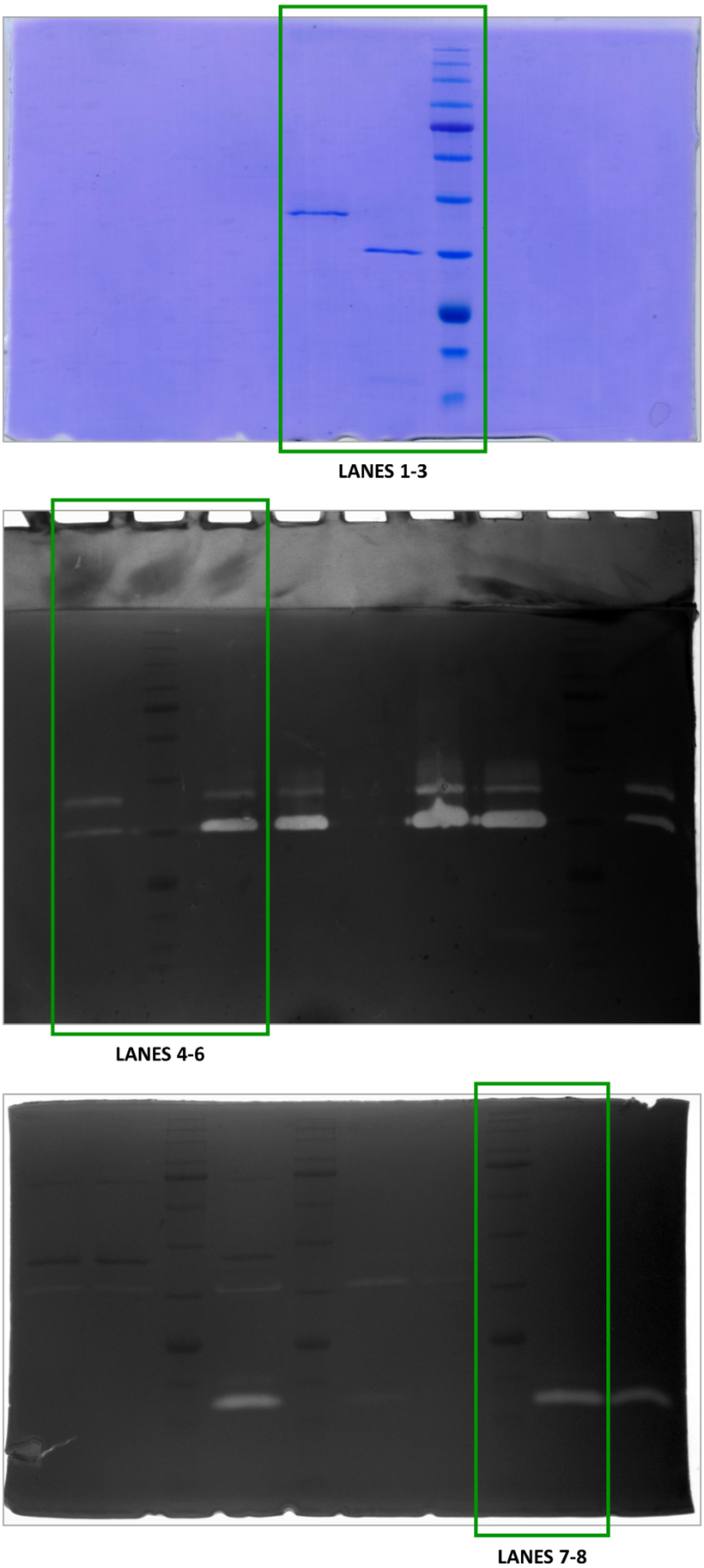

Extended Data Figure S15. SDS-PAGE analysis of complete CPL1_1-3 activation by trypsin (top panel; lanes 1-3), and zymogram analysis of both activated and non-activated CPL1 sample (middle panel; lanes 4-6). For reference, trypsin activity is shown at an apparent molecular weight of ~18 kDa (bottom panel; lanes 7-8). The full, uncropped SDS-PAGE gel and zymograms corresponding to **Figure 6A** are provided, with the cropped regions indicated by a red box for clarity.

Extended Data Figure S16:
Non-cropped gel images of Supplementary Figure S1A.

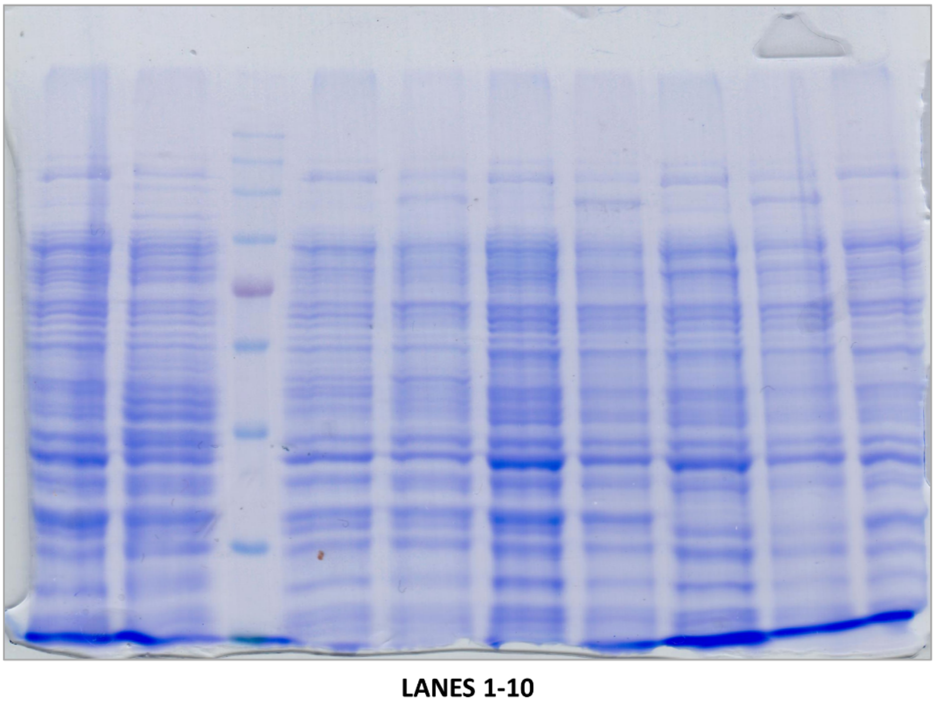

Extended Data Figure S16. SDS-PAGE-based expression analysis of full-length wild-type CPL1 (Q20–K1282; HExxH) cloned into vector pCri7a* in Escherichia coli strains BL21(DE3), Origami 2(DE3), Lemo21(DE3) and Rosetta(DE3). The full, uncropped SDS-PAGE gel corresponding to **Supplementary Figure S1A** is provided.

Extended Data Figure S17:
Non-cropped gel images of Supplementary Figure S1B.

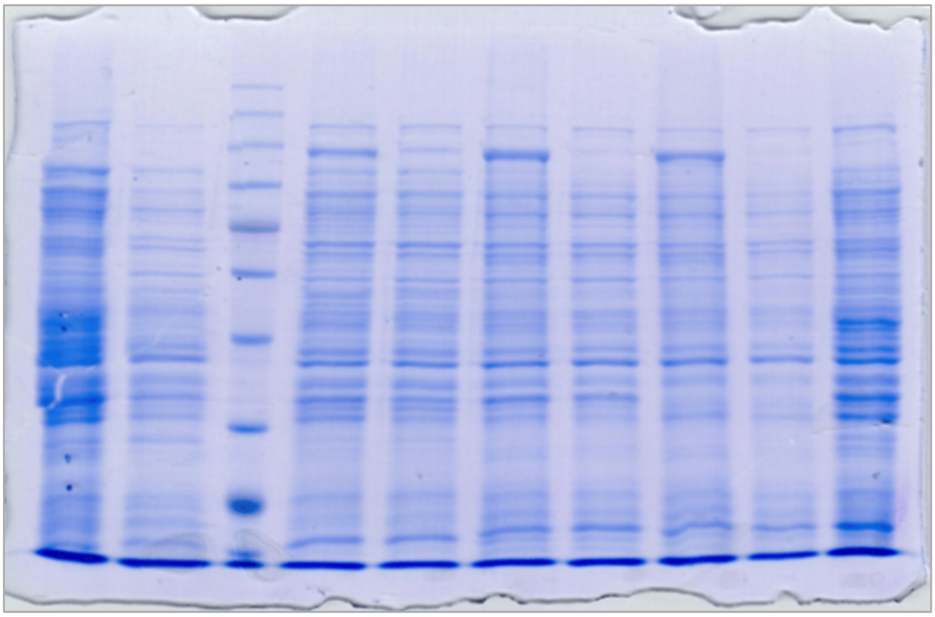

LANES 1-10

Extended Data Figure S17. SDS-PAGE-based protein solubility assessment of full-length wild-type CPL1 (Q20–K1282; HExxH) cloned into vector pCri7a* in Escherichia coli strains BL21(DE3), Origami 2(DE3), Lemo21(DE3) and Rosetta(DE3). The full, uncropped SDS-PAGE gel corresponding to **Supplementary Figure S1B** is provided

Extended Data Figure S18:
Non-cropped gel images of Supplementary Figure S1D.

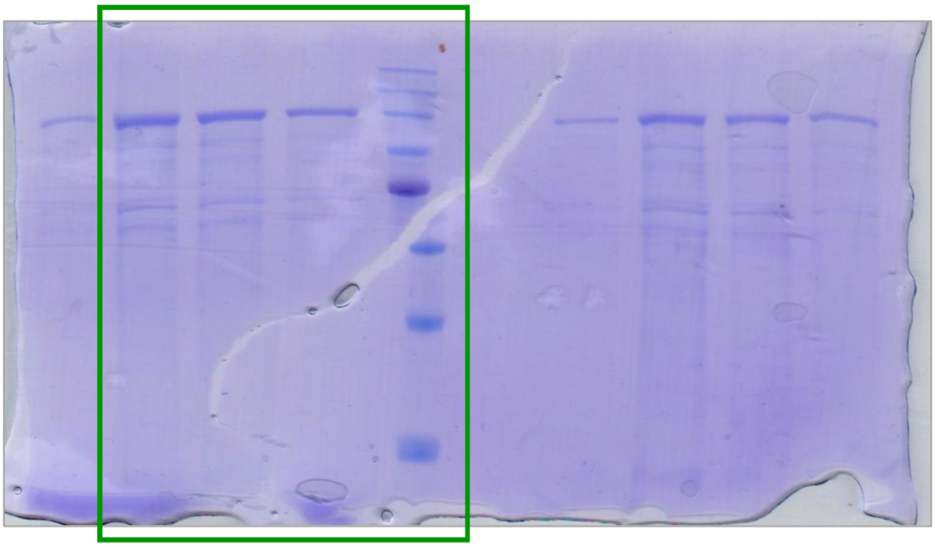

LANES 1-4

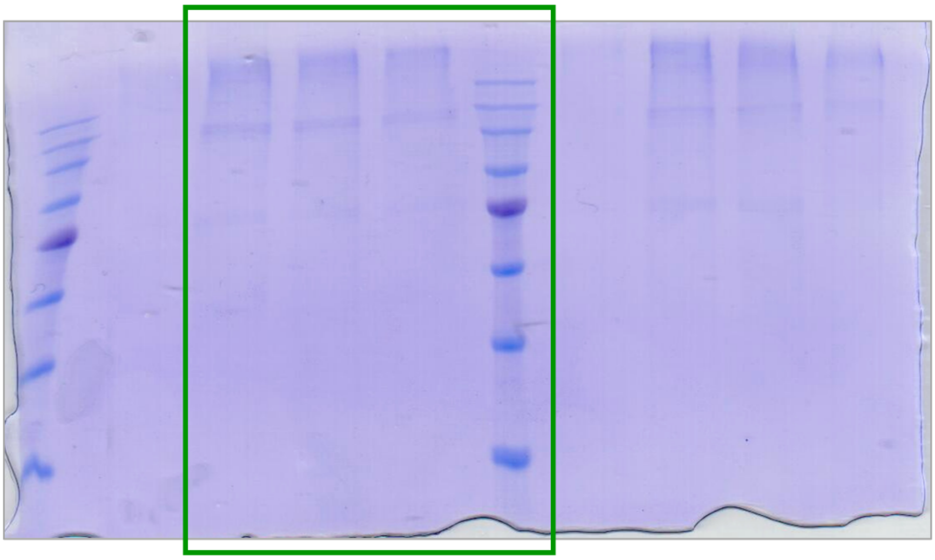

LANES 5-8

Extended Data Figure S18. SDS-PAGE analysis at both reducing (Top; lanes 1-4) and non-reducing (Bottom; lanes 5-8) conditions of full-length wild-type CPL1 (Q20–K1282; HExxH) after size exclusion chromatography. The full, uncropped SDS-PAGE gels corresponding to **Supplementary Figure S1D** are provided.

Extended Data Figure S19:
Non-cropped gel images of Supplementary Figure S2A.

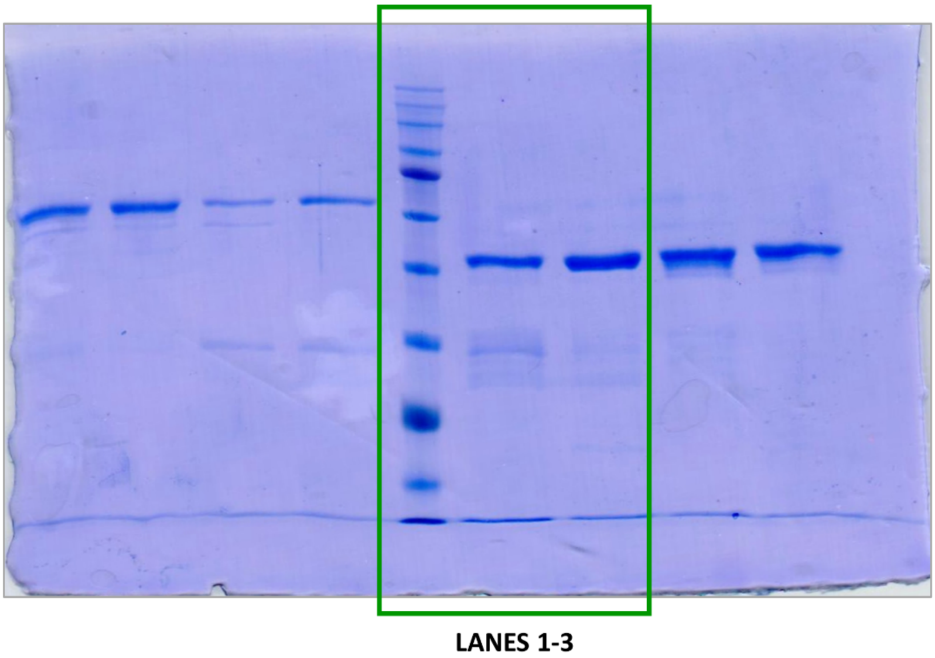

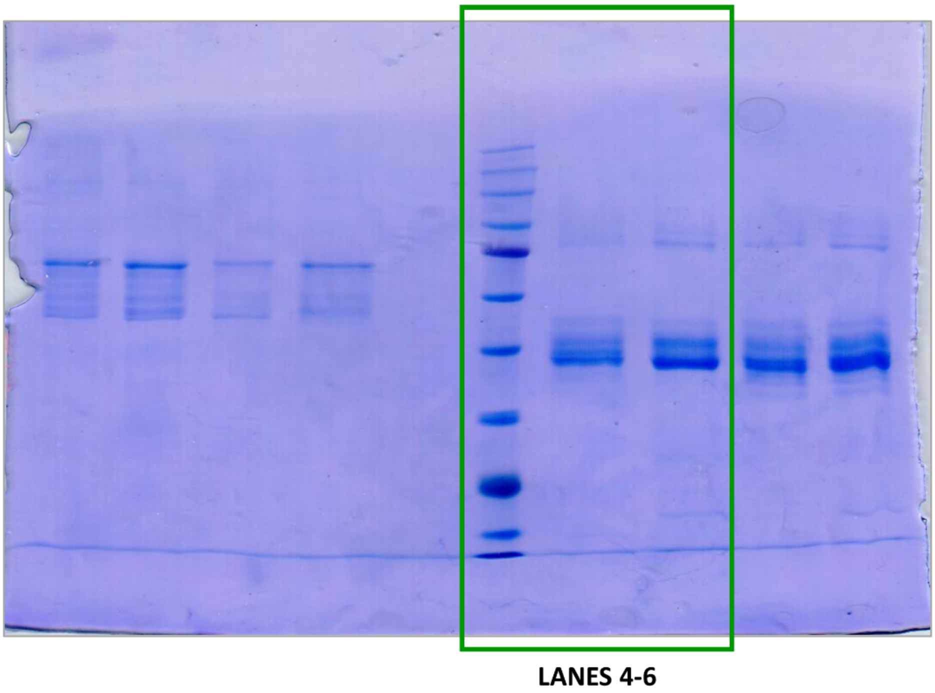

Extended Data Figure S19. SDS-PAGE analysis under reducing (top; lanes 1-3) and non-reducing (bottom; lanes 4-6) conditions of the C-terminally deletion constructs of wild-type CPL1_1-3 (HExxH). The full, uncropped SDS-PAGE gels corresponding to **Supplementary Figure S2A** are provided.

Extended Data Figure S20:
Non-cropped gel images of Supplementary Figure S2B.

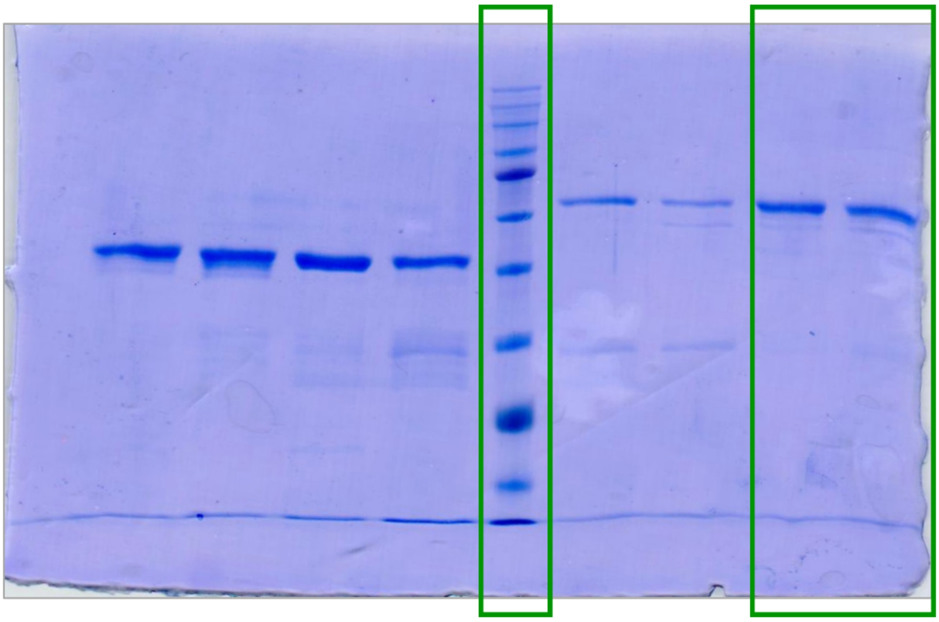

Same SDS-PAGE gel (mirrored) as used for Supplementary Fig. 2A.

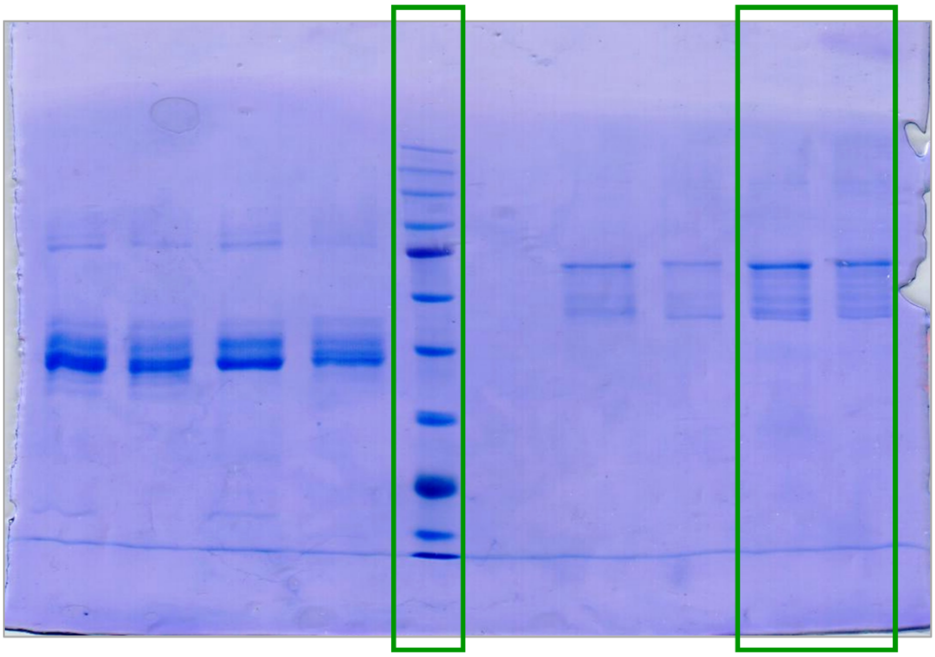

Same SDS-PAGE gel (mirrored) as used for Supplementary Fig. 2A .

Extended Data Figure S20. SDS-PAGE analysis under reducing (top; lanes 1-3) and non-reducing (bottom; lanes 4-6) conditions of the C-terminally deletion construct of CPL1_1-4 mutant E232A (HAxxH). The full, uncropped SDS-PAGE gels corresponding to **Supplementary Figure S2B** are provided.

Extended Data Figure S21:
Non-cropped gel images of Supplementary Figure S2C.

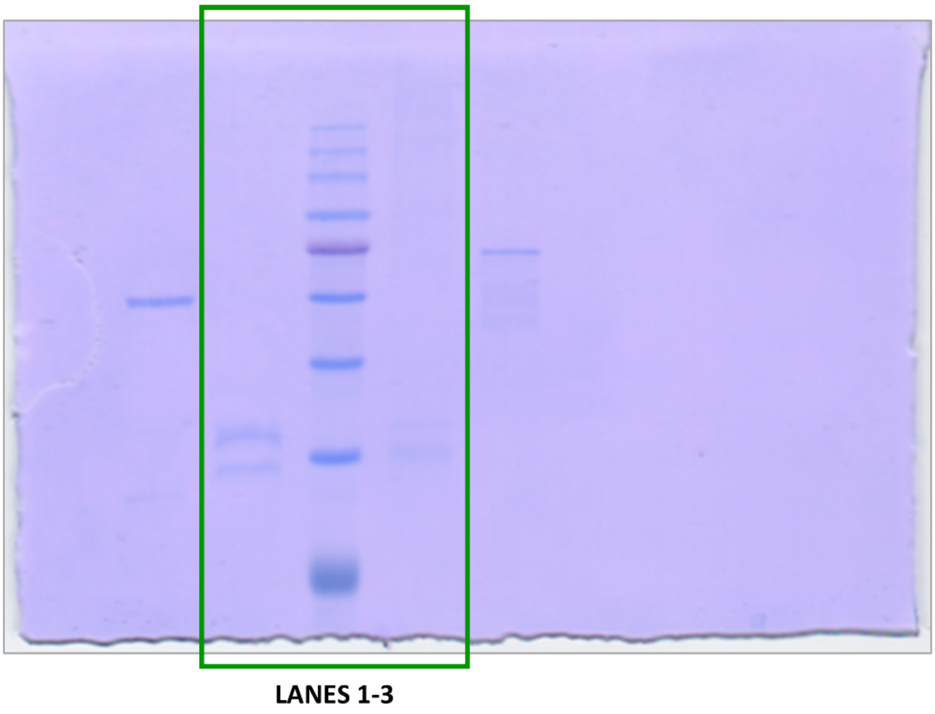

Extended Data Figure S21. SDS-PAGE analysis under reducing (left; lane 1) and non-reducing (right; lane 3) conditions of the C-terminally deletion construct CPL1_1-2 (HExxH). The full, uncropped SDS-PAGE gel corresponding to **Supplementary Figure S2C** is provided.
